# PD-L1 Nanobody Competitively Inhibits the Formation of the PD-1/PD-L1 Complex: Comparative Molecular Dynamics Simulations

**DOI:** 10.3390/ijms19071984

**Published:** 2018-07-07

**Authors:** Xin Sun, Xiao Yan, Wei Zhuo, Jinke Gu, Ke Zuo, Wei Liu, Li Liang, Ya Gan, Gang He, Hua Wan, Xiaojun Gou, Hubing Shi, Jianping Hu

**Affiliations:** 1 College of Pharmacy and Biological Engineering, Sichuan Industrial Institute of Antibiotics, Key Laboratory of Medicinal and Edible Plants Resources Development of Sichuan Education Department, Antibiotics Research and Re-evaluation Key Laboratory of Sichuan Province, Chengdu University, Chengdu 610106, China; sunbsxw123@163.com (X.S.); yan_xiao163@163.com (X.Y.); zuoke2015@outlook.com (K.Z.); liuwei@cdu.edu.cn (W.L.); lianglicdu@163.com (L.L.); Ganfcd@outlook.com (Y.G.); hegang@cdu.edu.cn (G.H.); 2 Ministry of Education Key Laboratory of Protein Science, Tsinghua-Peking Joint Center for Life Sciences, Beijing Advanced Innovation Center for Structural Biology, School of Life Sciences, Tsinghua University, Beijing 100084, China; zhuowei1989@163.com (W.Z.); gujinke@tsinghua.edu.cn (J.G.); 3 College of Mathematics and Informatics, South China Agricultural University, Guangzhou 510642, China; wanhua@scau.edu.cn; 4 Laboratory of tumor targeted and immune therapy, Clinical Research Center for Breast, State Key Laboratory of Biotherapy, Sichuan University and Collaborative Innovation Center for Biotherapy, Chengdu 610041, China; shihubing77@sina.com

**Keywords:** PD-1/PD-L1, monoclonal antibody, MD simulation, inhibitory mechanism, antibody design

## Abstract

The anti-PD-L1 monoclonal antibody (mAb) targeting PD-1/PD-L1 immune checkpoint has achieved outstanding results in clinical application and has become one of the most popular anti-cancer drugs. The mechanism of molecular recognition and inhibition of PD-L1 mAbs is not yet clear, which hinders the subsequent antibody design and modification. In this work, the trajectories of PD-1/PD-L1 and nanobody/PD-L1 complexes were obtained via comparative molecular dynamics simulations. Then, a series of physicochemical parameters including hydrogen bond, dihedral angle distribution, p*K*a value and binding free energy, and so forth, were all comparatively analyzed to investigate the recognition difference between PD-L1 and PD-1 and nanobody. Both _L_R113 (the amino acid residues in PD-L1 are represented by the lower left sign of L) and _L_R125 residues of PD-L1 undergo significant conformational change after association with mAbs, which dominates a strong electrostatic interaction. Solvation effect analysis revealed that solvent-water enhanced molecular recognition between PD-L1 and nanobody. By combining the analyses of the time-dependent root mean squared fluctuation (RMSF), free energy landscape, clustering and energy decomposition, the potential inhibition mechanism was proposed that the nanobody competitively and specifically bound to the β-sheet groups of PD-L1, reduced the PD-L1’s flexibility and finally blocked the formation of PD-1/PD-L1 complex. Based on the simulation results, site-directed mutagenesis of _N_D99 (the amino acid residues in Nano are displayed by the lower left sign of N) and _N_Q116 in the nanobody may be beneficial for improving antibody activity. This work offers some structural guidance for the design and modification of anticancer mAbs based on the structure of the PD-1/PD-L1 complex.

## 1. Introduction

Cancer has become one of the major threats to human life and health [1]. According to the 2014 report of the International Agency for Research on Cancer (IARC), about 1.4 million new cancer patients were added from 2008 to 2012 and it was estimated that the number of cancer cases will increase to 25 million in the next 20 years [2]. Currently, the primary methods for cancer treatment are surgical resection, radiotherapy, chemotherapy, immunotherapy and so on [3,4,5,6]. Surgical resection temporarily removes the lesion, which cannot be completely removed due to the strong metastatic characteristic of tumor cells. Radiotherapy and chemotherapy both kill tumor cells accompanied by accidentally injuring normal cells associated with the poor targeting, being prone to drug resistance. Immunotherapy targets are not cancer cells but the body’s immune system, such as immune checkpoint molecules, which is different from the traditional treatment strategy. The particularity of the target growth environment can effectively decrease the side effects of drugs. Extensive tests [7] demonstrated that immunotherapy enhances overall survival of patients. In 2013, Science Journal listed tumor immunotherapy as “Annual-Top Ten Scientific Breakthroughs [7]”.

**Figure 1:**
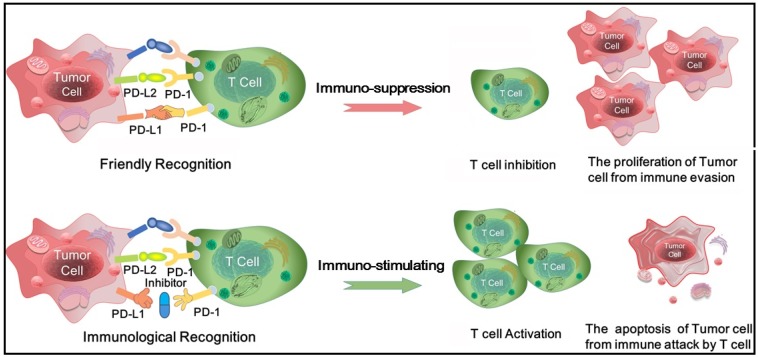
The physiological process for T cells recognizing and killing tumor cells with the aid of the PD-1/PD-L1 complex.

The main target of immunotherapy is the immune checkpoint molecules which are a number of inhibitory signaling pathways present in the immune system [8]. In vivo, the immune checkpoint is properly to maintain immune tolerance by regulating the intensity of the autoimmune response. Nevertheless, when the body is attacked by tumor, the immune checkpoint is activated that inhibits autoimmunity and promotes tumor cells to grow and escape [9]. Currently, two immunological checkpoint drugs which aim at cytotoxic T-lymphocyte-associated antigen 4 (CTLA-4) and programmed death-1/programmed death ligand1 (PD-1/PD-L1) have been approved for marketing. CTLA-4 is the first immunological checkpoint molecule used in immunotherapy with sensational clinical efficacy, howbeit CTLA-4 inhibitors have been reported to have some side effects [10,11,12]. PD-1/PD-L1 inhibitors have a lot of advantage with short half-life, weak binding, less toxicity and effectively curb tumor merisis and reproduction [10]. PD-1 is a co-stimulatory molecule that is induced on the surface of activated T cells. It is composed of extracellular immunoglobulin variable regions (IgV), transmembrane and intracellular domains. PD-1 has two natural ligands PD-L1 and PD-L2 that expressed in both tumor cells and antigen-presenting cells. PD-L1 is more extensively expressed than PD-L2 and grows in a variety of tumor cells, thus PD-L1 is often used as a target for anti-cancer drug design. PD-1/PD-L1 pathway reverses the activated T cell, decreases its activity and induces its apoptosis. On the contrary, PD-1/PD-L1 inhibitors effectively block the PD-1/PD-L1 pathway and enhance the activity of T cell, thus achieving the effect of tumor immunotherapy [13,14,15] (see Figure 1 in detail).

Monoclonal antibodies have numerous merits, such as high specificity, few side effects and being prepared in massive manufacture. The current clinical applications of PD-1/PD-L1 inhibitory drugs are basically monoclonal antibodies [15], most of which are PD-1 inhibitors and anti-PD-L1 ones are less. In fact, targeting PD-L1 antibodies were able to avoid adverse reaction associated with PD-L2 [16]. Up to now, there is no report on the complete co-crystal structure of PD-1/PD-L1 with ligands which results in slow progress in the study of small molecule inhibitors based on PD-1/PD-L1 structure, with no small molecule inhibitors entering the clinical stage [17]. The discovery of molecular recognition and inhibitory mechanism for PD-1/PD-L1 monoclonal antibodies will obviously promote development of small molecule drugs.

Owing to the key role and obvious curative effect of monoclonal antibody in clinical application, many research groups have emphasized on this hot scientific region. Lately, Na et al. [18] obtained a complex of pembrolizumab Fab region and PD-1 by X-ray crystallography, enriching the structural bioinformatics. Lee [19] also determined the similar crystal structure with higher resolution (2.0 Å). In vitro experiments showed that the toxicity of PD-L1 antibodies was low [20]. In the field of theoretical research, Zak et al. [21] found that the antibodies (i.e., pembrolizumab and nivolumab) mainly interact with the CC’FG region of PD-1/PD-L1. Moreover, Ahmed et al. [22] performed conventional molecular dynamics (cMD) and accelerated MD (aMD) simulations and found that the conformation changes of BC loop and C’’D loop were largest when PD-L1 was combined with antibodies. Liu [23] sought out the epitopes of PD-1 by MD simulation and found that three of the key residues (i.e., N66, K78 and A132) participate in the recognition association with of PD-1/PD-L1. Hao’s group [11] proposed that PD-L1-based antibodies mainly bind to the four regions of PD-L1 (CC’FG) and FG, which is particularly important [19].

**Figure 2:**
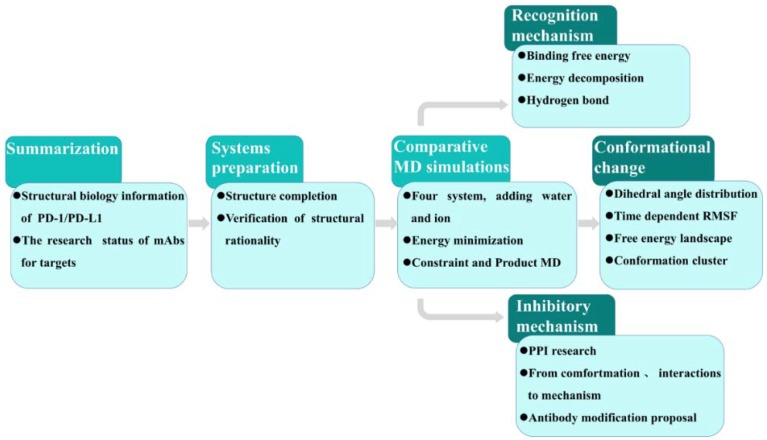
Protocol for the prediction of the molecular mechanism inhibiting PD-1/PD-L1 induced by PD-L1 nanobody.

The above research work promoted the development of antibody drugs but further exploration is required for understanding the inhibition mechanism of monoclonal antibody targeting PD-L1. The purpose of this work is basically to explore the conformation and flexibility of PD-L1 after identifying nanobody and elucidate the recognition mechanism of nanobody with PD-L1. Figure 2 shows the research protocol in this work. It is composed of (1) summarized progress of structural information of PD-1/PD-L1 and its antibody inhibitors; (2) completing and verifying the target configuration used as the initial structure for the subsequent MD simulations; (3) obtaining trajectories of four systems by water-containing long-time MD simulations; (4) series analyses (including hydrogen bond, binding free energy and energy decomposition, dihedral angle, root mean squared fluctuation (RMSF), free energy landscape (FEL) and conformational cluster analysis) for exploring conformational change/molecular recognition/possible inhibitory mechanism and providing antibody modification proposals. This work is not only helpful in the development of antibody drugs based on the structure of PD-1/PD-L1 but also has application value in the clinical antibody medicine.

## 2. Results and Discussion

### 2.1. Structural Biology Survey of PD-1/PD-L1

Up to now, 26 three-dimensional structures of PD-1 or PD-L1 have been reported in protein data bank (PDB: www.rcsb.org). Table 1 lists the available structural information of PD-1 and PD-L1 so far. As seen from Table 1, the reported structures of PD-1_apo and PD-L1_apo consist of the complete amino acid sequence and the protein aggregates include monomers and dimers. In addition, all the structures have a relatively high resolution of less than 2.5 Å, among them the highest resolution is 5C3T protein (i.e., 1.8 Å) and 3RRQ belongs to human PD-1 with a resolution of 2.1 Å. Five complex structures of intact human PD-1 with antibodies were published by Lee et al., which was helpful to observe the binding pattern between PD-1 and antibody inhibitors. On the basis of previous studies, this work aims to obtain the optimized binding and motion modes of PD-L1 with antibodies, reveal the inhibitory mechanism of antibody and finally provide structural guidance for modification of antibody.

Among the reported five crystal structures of PD-L1 complexed with antibody, 5JDS has the highest resolution of 1.7 Å. Its PD-L1 sequence is complete and the substrate is the newest antibody drug (i.e., nanobody KN035-Fc). It is worth mentioning that PD-L1 antibodies have less immune-related toxicity than anti-PD-1 therapy because of the higher selectivity and the lower immune response in the tumor microenvironment [16,36,37,38]. Zak et al. [34] first presented the crystal structure of PD-L1 complexed with small molecule agents and proposed that small molecule inhibitors could promote the dimerization of PD-L1 and destroyed the formation of PD-1/PD-L1 complex. In the term of PD-1/PD-L1 crystal structure, the primary 3BIK and 3SBW were both murine PD-1/human PD-L1 complex with some deficiencies in anticancer drug development. Fortunately, 5IUS and 4QKZ deposited recently, both belong to human PD-1/PD-L1 complexes. According to Table 1, all the 3RRQ, 5C3T, 5JDS and 4ZQK structures were selected for the subsequent comparative MD simulations to investigate conformational changes, molecular recognition and antibody modification and so forth.

**Figure 3:**
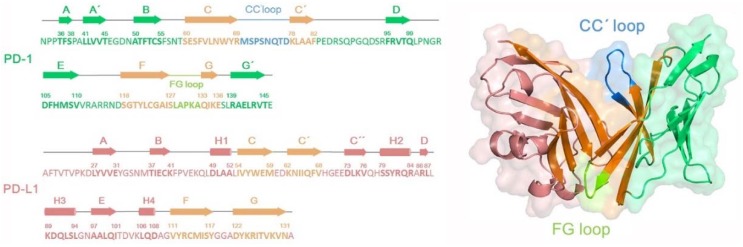
The secondary structure sequences and corresponding three-dimensional structures for the PD-1 and PD-L1 systems.

It is necessary to briefly understand sequence and structure of the PD-1/PD-L1 system (see Figure 3) prior to investigating molecular recognition between PD-L1 and antibody molecules, as well as the rational PD-L1 antibody modification. As shown in Figure 3, the secondary structure of PD-1 contains a large number of β-sheets, among which the orange parts are involved in the binding of PD-L1 or antibodies with the help of CC’ (in blue) and FG loops (in light green). The CC’ loop region undergoes conformational rearrangement after binding PD-L1, while the conformation of FG loop is rather conservative favoring the structural maintenance [12]. As for PD-L1, the secondary structure consists of 4 α-helices and 9 β-sheets. C/C’/F/G regions (in orange) of PD-L1 are important for binding PD-1 or antibodies. It is worth mentioning that PD-1 and PD-L1 comprise a group of disulfide bonds, such as C54-C123 and _L_C40-_L_C114, which plays a key role in maintaining the stable interactions among β-sheet groups and improving the rigidity of the whole molecule [12].

### 2.2. Development of Antibodies Targeting PD-1/PD-L1

PD-1/PD-L1 is one of the most potential targets for immunotherapy. Antibody drugs targeting PD-1/PD-L1 have been utilized for clinical application. Antibody drugs have numerous advantages, such as high specificity, less damage to normal cells and curbing proliferation of most malignant tumors. Table 2 lists the research progress of PD-1/PD-L1 mAbs. PD-1/PD-L1 antibodies are divided into three classes according to different targets (including PD-1, PD-L1 and PD-L2). In particular, the PD-1 antibodies nivolumab [39] and pembrolizumab [40] were approved by the FDA for treatment of non-small cell lung cancer (NSCLC) in 2015 [17,41,42]. Atezolizumab [43], a new Roche type anticancer drug, was the first PD-L1 antibody approved in 2016 as second-line treatment NSCLC. The reported anti-PD-L1 mAbs including BMS-936559, durvalumab, avelumab, KN035-Fc, all have passed Phase I clinical trials for advanced solid tumors [17,30]. Currently, there are few studies on PD-L2 antibodies. The PD-L1-based mAbs showed higher affinity to the target relative to PD-1-based and PD-L2-based antibodies. For example, the IC_50_ value of KN035-Fc was 5.25 nM, easily penetrating large tumor cells. Previous studies have mainly involved biological experiments on mABs’ activity; nevertheless, some important scientific issues including molecular recognition, conformational change, motion mode of antibodies and receptors are not yet clear, leading to a slight deficiency in the theoretical basis for antibody modification. Elucidating the interactions between PD-L1 and its antibody is beneficial to the development and modification of PD-L1 mAbs.

### 2.3. Structure Complement of PD-1

MD simulations were carried out for the five systems (i.e., PD-1_apo, PD-L1_apo, Nano, PD-1/PD-L1 and Nano/PD-L1), in which the residues D85-D92 of PD-1 was not resolved. The missing residues were complemented with Discovery Studio 2.5 package [44]. After structural completion, the structural rationality of PD-1 was evaluated with the Ramachandran distribution and probability density function (PDF). The Ramachandran plot was able to evaluate the torsion degree of C_α_–C and C_α_–N bonds within peptide bonds of proteins and indicated the allowable and prohibitive conformations of residues. PDF reflects on bond length, angle, dihedral angle and other geometric properties of the constraints, that is, the smaller the PDF value, the more credible the model.

Figure S1 shows the PDF values and Ramachandran plot of the complemented PD-1 system. In Figure S1A, the blue color indicated the conformational optimal region illustrating that the more amino acids appear in this region, the more reliable the structure established. The purple region represented the conformational allowed area and the remainder was the forbidden one. By observation, the dihedral angle distributions of all residues were reasonable. Specifically speaking, 98.2% residues of PD-1 model locate in the conformational optimal region (in blue) and 1.8% residues in the conformational allowed area (in purple). In Figure S1B, the great majority of residues possess low PDF data apart from E46 and G47, which were far from the active pocket and had little effect on subsequent MD simulations. 

**Figure 4:**
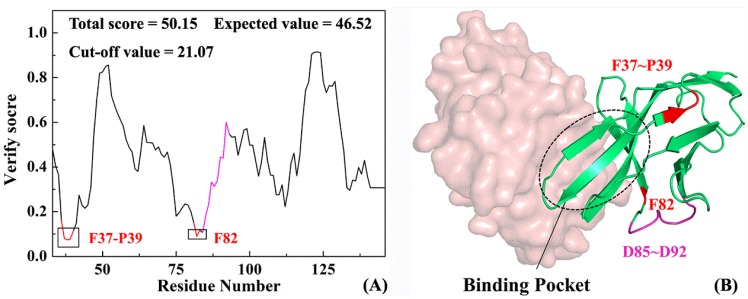
Verify score (**A**) and corresponding 3D structure (**B**) of the PD-1 system. Red indicates the region with high verify score that the modeling result is slightly poorer and pink shows the missing residues D85–D92.

Profile-3D is an evaluation program that applies verify score function to evaluate the matching degree between a structural model and amino acid sequence [45]. The higher the verify score, the more reliable the protein structure. Figure 4A shows the verify score on each residue in PD-1 after completion with a total score of 50.15, an expected value of 46.52 and a cut-off value of 21.07. The actual value is close to the expected one and is higher than the critical value, which indicates that the structure obtained by homologous modeling is reasonable. In addition to the verify scores of residues F37-P39, F82 less than 0.1, values of 97% residues are more than 0.1, which at the residue level proves the reliability of protein homologous modeling. Figure 4B shows a three-dimensional model of the PD-1 complementary structure, in which the green and brown indicate PD-1 and PD-L1, respectively. Fortunately, very few unfavorable modeling region (in red) with low verify score is far from the active pocket, exerting less influence on the association between PD-1 and its partners (such as PD-1 or antibodies etc.). In sum, the reliable full-length PD-1 model is the basis of exploring the following structure-function relationships and molecular recognition.

### 2.4. The Reliability of Molecular Dynamics Simulations

The potential energy of the five systems (i.e., the PD-1_apo, PD-L1_apo, Nano, PD-1/PD-L1 and Nano/PD-L1 systems) approached the equilibrium after 2.52 ns which showed the five systems are relatively stable in MD simulations. After 30 ns, the root mean square deviations (RMSDs) of PD-1_apo, PD-L1_apo and PD-1/PD-L1 tended to be stabilized with about 1.80 ± 0. 20, 1.71 ± 0. 22 and 2.1 ± 0.24 Å, respectively. Comparatively, the RMSD of PD-1/PD-L1 is higher, which is mainly due to the short half-life in its natural state and low affinity of PD-1 with PD-L1 [25]. It has also been disclosed that the weak binding of PD-1 with its endogenous ligand PD-L1 provides the possibility of competitive inhibition for subsequent PD-1/PD-L1 antibody drug design.

Figure S2A shows the distributions of root mean square fluctuation (RMSF) of C_α_ atoms in PD-1/PD-L1, PD-1_apo and PD-L1_apo and the correlation between simulated B-factor values and experimental ones of PD-1/PD-L1 which suggests that the obtained MD trajectories are reliable and suitable for the subsequent analyses.

**Figure 5:**
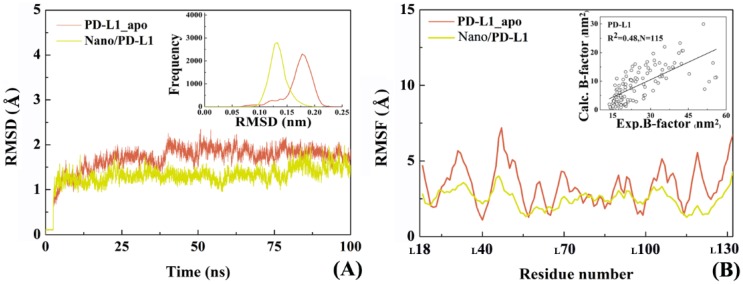
Overall conformational analyses of the two investigated systems. (**A**) The root mean square deviations (RMSDs)variety of C_α_ atoms of PD-L1_apo (coral) and Nano/PD-L1 (yellow) over time, respectively; (**B**) The root mean squared fluctuation (RMSF)distribution of Cα atoms of PD-L1_apo (coral) and PD-L1 (yellow) in the antigen-antibody complex, respectively.

Subsequently, the overall conformational properties were analyzed for the Nano/PD-L1 system. Figure 5 comparatively shows the change of C_α_ atomic RMSD in PD-L1_apo and Nano/PD-L1 over time. The C_α_ atomic RMSD of PD-L1 from equilibrium MD trajectories reduce from 1.71 ± 0.22 Å to 1.30 ± 0.18 Å after the recognition by nanobody, however the RMSD values of the Nano maintained at 0.80 + 0.18 Å during the simulation. Compared with the RMSD value of PD-1/PD-L1 (2.10 ± 0.24 Å), nanobody and PD-L1 can form a more stable complex than the endogenous PD-1/PD-L1 complex. It is observed that the RMSF distribution of PD-L1 in the two systems (i.e., PD-L1_apo and Nano/PD-L1) are similar, notwithstanding, nanobody significantly weakens the flexibility of PD-L1_apo of which the RMSF value fluctuated around 3 Å. Especially, the local flexibility of β-sheets (_L_I54-_L_M59, _L_K62-_L_V68, _L_V111-_L_S117 and _L_D122-_L_N131) in the Nano/PD-L1 is much lower than that in PD-1/PD-L1. According to the difference between the mean RMSD values calculated by the equilibrium section of the trajectories (20–100 ns) of PD-1/PD-L1 and Nano/PD-L1, the latter is much more stable than the former in the simulation process. In this work, the trajectories of 20–100 ns in each system were selected for the subsequent conformational analyses. The radius of gyration analysis, on the other hand, also hinted the volume of Nano/PD-L1 has not changed substantially (see Figure S3). As shown in Figure 5, in a nutshell, two viewpoints can be gained. First, the association of nanobody leads to the global and local conformational change of PD-L1 and the conformational change and motion mode both are necessary to be analyzed in detail. Then, the satisfactory correlation between the experimental B factors and the calculated ones (*R*^2^ = 0.48, *N* = 115) also suggests that our productive MD is rational and dependable. 

### 2.5. Molecular Motion Analysis

**Figure 6:**
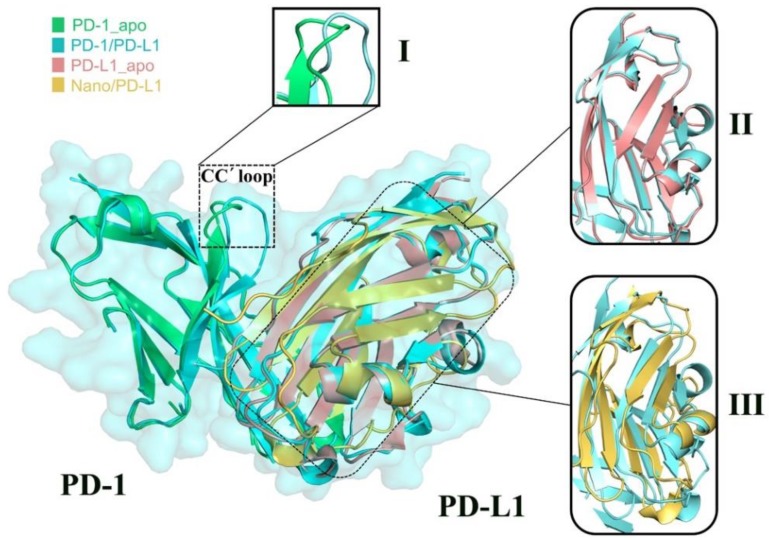
Superimposition of crystal structures for the PD-1_apo (in green), PD-1/PD-L1 (in blue), PD-L1_apo (in brown) and Nano/PD_L1 (in yellow) systems. Region I shows the structural differences of the CC’ loop compared to the PD-1_apo and PD-1/PD-L1 systems. Region II and III show the structural difference of β-sheet groups between PD-L1_apo and PD-1/PD-L1, as well as between Nano/PD-L1 and PD-1/PD-L1.

Four PDB crystal structures (i.e., 3RRQ, 4ZQK, 5C3T and 5JDS) were superimposed together, represented in green, blue, pink and yellow, respectively (see Figure 6). The good superimposition indicates that PD-1 and PD-L1 molecules have high stability and rigidity. Three other features can also be observed: (1) The CC’ loop of PD-1 undergoes a significant conformational rearrangement [12] induced by the association of PD-L1; (2) After binding PD-1, the structural change of β-sheet groups of PD-L1 is small, reflecting its inherent structural rigidity; (3) The β-sheet groups of PD-L1 show obvious structural difference after binding various partners such as PD-1 and nanobody. Based on the above observations, it is suggested that the β-sheet groups of PD-L1 exhibited high conservation in association with the endogenous PD-1 partner but the binding of the nanobody has a great influence on this regional structure, which may be related to the nanobody’s inhibitory mechanism. It is well known that the conformational change of drug targets has a certain intrinsic relationship with the following drug design, thus then molecular motion analysis was performed.

**Figure 7:**
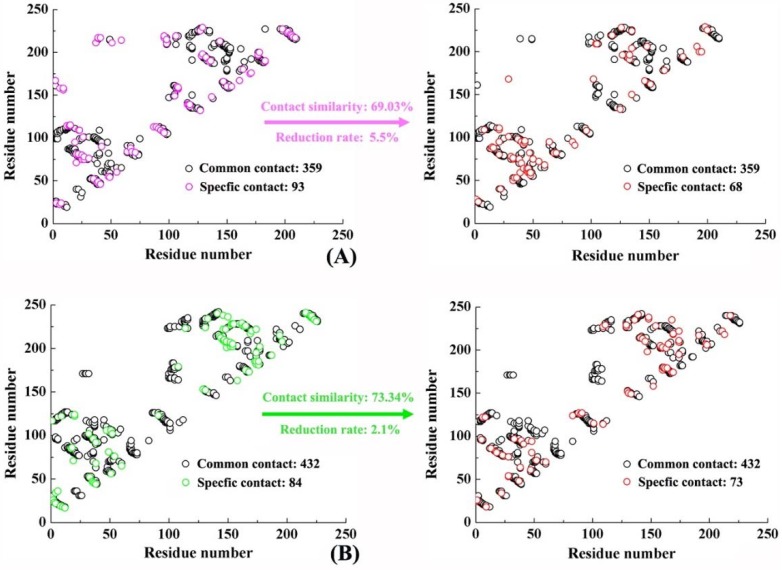
Residue contact maps of the PD-1/PD-L1 (**A**) and Nano/PD-L1 systems (**B**).

A residue contact map is an effective method used for describing the conformational change of biomolecules [46]. If the distance between two residues in a biomacromolecular system is less than 4.5 Å, then the two residues can be said to have a connection [47]. The difference of contact residues between the initial (at 0 ns) and the final (at 100 ns) structures in PD-1/PD-L1 and Nano/PD-L1 was investigated. The initial structures of PD-1/PD-L1 and Nano/PD-L1 have 452/516 residual contacts, which is reduced to 427/505 in the final structure. There is 359/432 same residual contact in the initial and final conformations, while the special residual contact is 93/68 and 84/73, respectively (See Figure 7). To describe the conservativeness of the residual contacts and the extent of expansion and relaxation of the complex, two parameters, contact similarity and reduction rate, are defined in this work. The contact similarity is computed by the common contacts within both initial and final structures divided by the total contacts covering common and specific contacts in both the first and last structures in MD simulation. Moreover, the reduction rate is calculated by the distinction between the number of specific contacts in all conformations divided by the total number of contacts including common and specific contacts in the initial structure. The contact similarities and reduction rates of PD-1/PD-L1 and Nano/PD-L1 systems are 69.03%/73.34% and 5.5%/2.1% respectively. PD-1/PD-L1 and Nano/PD-L1 complexes both showed strong conservation but the latter are more obvious. Moreover, both systems exhibit subtle expansion with the small reduction rate, which is consistent with previous RMSD analysis. From Figure 7, two other features can be found. (1) The similarities in the distribution of contact residues between PD-1/PD-L1 and Nano/PD-L1 may be related to the fact that nanobody is derived from IgG1 which is the same to PD-1 [30]; (2) The contact residues of _L_I54-_L_M59, _L_Y112-_L_M115 and _L_D122-_L_R125 in PD-1/PD-L1 (corresponding to 37–42, 95–98 and 105–108 in Figure 7A) gradually disappear over MD simulation time. All three residual fragments are located in the β-sheet group recognition region, revealing a higher conformational flexibility in this region which agrees well with the previous RMSF analysis (see Figure S2C).

Furthermore, the vmdICE program [48] was employed to observe the RMSF over time for both the PD-1/PD-L1 and Nano/PD-L1 systems. In Figure S4, the value of RMSF is high in orange area, while in blue area the value of RMSF is low. The black dotted line suggests the region of β-sheet groups of PD-L1 (CC’: _L_I54-_L_V68; FG: _L_V111-_L_N131). It can be seen that the RMSF values always maintain a high level during the 100 ns simulation in the PD-1/PD-L1 system (see Figure S4A). The flexibility of these highly flexible regions of PD-L1 was significantly reduced after recognition by antibodies. For instance, nanobody significantly decreases the flexibility of CC’ and FG regions and weakens their mobility. The highly flexible CC’ and FG domains are the key factors for the recognition of PD-1 with PD-L1. Then, we speculated that nanobody might partially undermine the association of PD-1 with PD-L1and activate T cells to exert the biological function suppressing tumor cell growth.

The RMSF value of each residue reflects its flexibility, however, contains little structural information. Hence, DSSP program [49] was used to predict the secondary structures of PD-1_apo, PD-L1_apo, PD-1/PD-L1 and Nano/PD-L1. The conformation of each system was collected every 50 ps and 2000 snapshots were obtained in total. The result was shown in Figure S5. It can be found that on the whole, all systems are mostly composed of many β-sheet regions. The secondary structure of PD-1 and PD-L1 did not change obviously before and after the combination, which was consistent with the superimposition analysis. However, the local secondary structure of _L_D90-_L_Q100 in PD-L1_apo and PD-1/PD-L1 varied distinctly from that of Nano/PD-L1 in the simulations. In PD-L1_apo and PD-1/PD-L1, _L_D90-_L_Q100 composes α-helix. Nevertheless, in the Nano/PD-L1, _L_D90-_L_Q100 formed 3-helix, which may be due to the redistribution of interactions (i.e., salt bridge) within this region.

**Figure 8:**
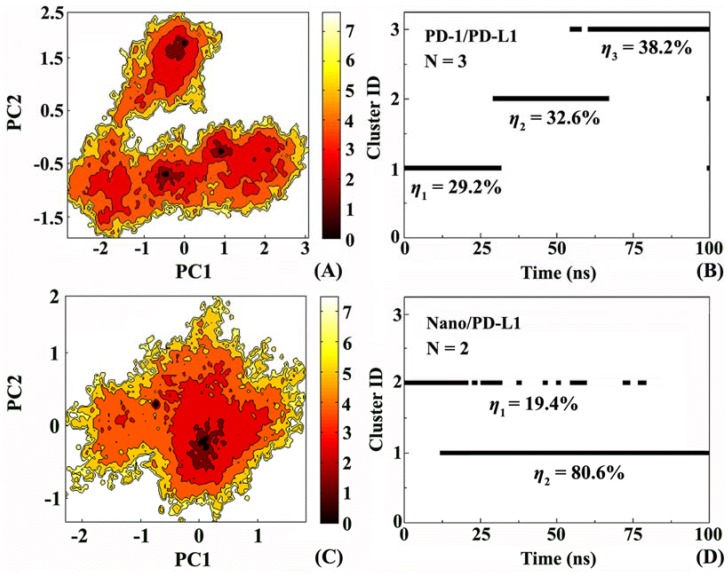
Free energy landscape and the corresponding conformational cluster analyses for the PD-1/PD-L1 (**A**,**B**) and Nano/PD-L1 (**C**,**D**) systems. PC1 and PC2 respectively represent principal component 1 and 2.

In order to understand the mobility of the system more accurately, free energy landscape and the corresponding conformational cluster analysis were performed. Figure 8A,C display the free energy surfaces of PD-1/PD-L1 and Nano/PD-L1 at 300 K, respectively. The darker areas indicate higher conformation distribution probability, relating to low free energy value. PD-1/PD-L1 and Nano/PD-L1 have 3 and 2 relative independent low free energy regions, respectively, representing the most stable conformation states in MD simulation. Unexpectedly, Nano/PD-L1’s molecular motility is more than that of endogenous PD-1/PD-L1 complexes. Furthermore, the shapes of the two free energy surfaces are significantly different and the conformational transition space of PD-1/PD-L1 is larger than that of Nano/PD-L1. It reveals that the binding potential of PD-L1 with nanobody is even higher than that with its endogenous ligand PD-1, embodied by the great favorable inhibitory activity of nanobody (IC_50_ = 5.25 nM). 

To discuss the details of the conformational changes for the PD-1/PD-L1 and Nano/PD-L1 systems, the structure clustering analyses were carried out. RMSD = 0.17 Å was set as the threshold and the clustering results of MD simulation trajectories of the two complexes are given in Figure 8B,D. PD-1/PD-L1 has 3 clusters which basically include the first cluster before 30 ns, the second cluster between 30 and 66.5 ns and the third cluster mainly after 61.5 ns. Nano/PD-L1 possesses only two clusters and the dominant conformation is after 12.5 ns, with conformational probability of 80.6%. In combination with the results of cluster and free energy landscape analyses, it is found the nanobody binding decreased the mobility exhibiting more stable than PD-1/PD-L1.

**Figure 9:**
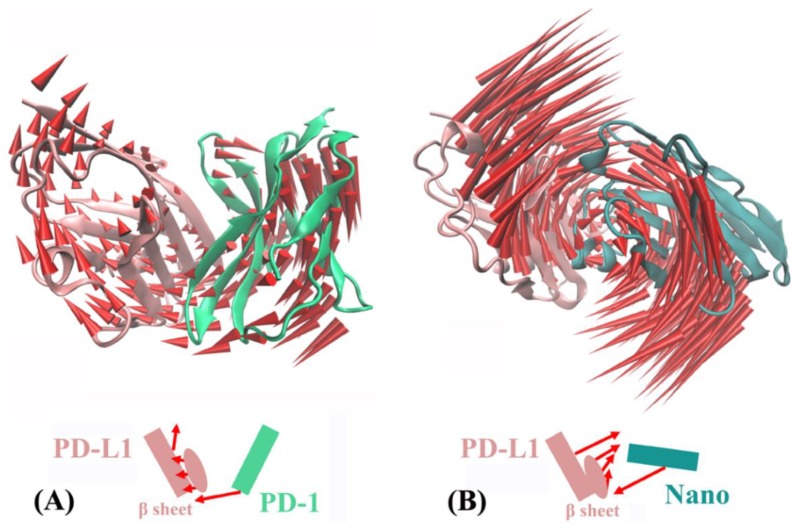
The first slow motion mode of the PD-1/PD-L1 (**A**) and Nano/PD-L1 (**B**) systems.

Based on principal component analysis, Figure 9 shows the overall motion direction and amplitude for the PD-1/PD-L1 and Nano/PD-L1 systems. The two systems have a slightly similar slow motion mode, with substantial rotation movements along the axis between PD-L1 and its partners (i.e., nanobody and PD-1). Nevertheless, there are some noticeably difference. Nano/PD-L1 has a bit greater motion amplitude than PD-1/PD-L1. Nanobody induces the conformational change of PD-L1, resulting in a more stable structure of Nano/PD-L1 (see Figure 8C,D). The motion direction of PD-L1 in two systems is evidently different. In PD-1/PD-L1, PD-L1 keeps away from PD-1 to some extent and the motion direction of β sheets is the same to that of PD-1. While in Nano/PD-L1, PD-L1 and nanobody are close to each other. The alteration of motion mode is helpful to the binding between PD-L1 and nanobody.

After analyzing the overall movement of PD-1/PD-L1 and Nano/PD-L1, we focused on the conformational change of the local binding regions, calculating the distributions of the dihedral angles *φ* and *ψ* for the seven key residues (i.e., _L_I54, _L_Y56, _L_R113, _L_M115, _L_A121, _L_Y213 and _L_R125) in PD-L1. Figure S6A,B represent the dihedral distribution of key residues in the β-sheet groups for the PD-1/PD-L1 and Nano/PD-L1 systems, respectively. When PD-L1 binds with different partner (i.e., PD-1 and nanobody), the dihedral angle distribution of key residues in the contact interface just exhibits subtle differences, which mainly lies in two points. First, the association with nanobody makes the dihedral angle of _L_Y56 slightly widely distributed, which may be induced by the non-polar interactions of the opposite benzene ring of _N_F101. In addition, the conformational transition space of _L_R113 and _L_R125 tends to decrease. In fact, the previous biological experimental data showed that the mutations of _L_Y56 and _L_R113 can reduce the binding affinity of PD-L1 with nanobody [30] and the analyses of two-dimensional dihedral angles distribution also prove that _L_Y56 and _L_R113 both are essential for PD-L1 to exert its biological activity.

**Figure 10:**
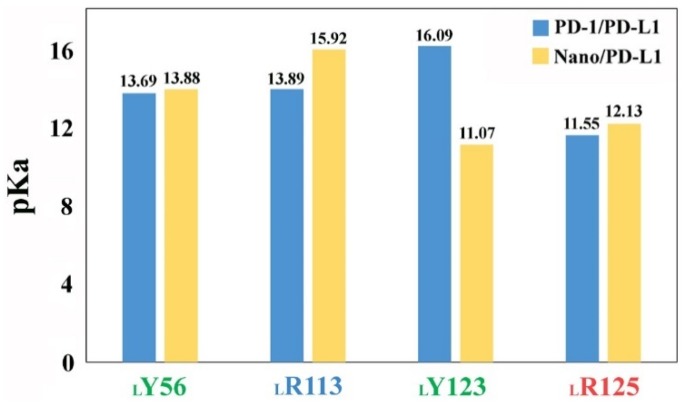
p*K*_a_ values of acidic and basic residues in the binding regions for the PD-1/PD-L1 and Nano/PD-L1 systems.

To investigate the change of the partial charge distribution in the binding domain between nanobody and PD-L1, the p*K*_a_ values of _L_Y56, _L_R113, _L_Y213 and _L_R125 were calculated with PROPKA [50] (see Figure 10). The p*K*_a_ value of residue side chain is an important indicator for evaluating enzyme activity and protein stability. As shown in Figure 10, the association of nanobody slightly increases the p*K*_a_ values of _L_Y56, _L_R113 and _L_R125 in PD-L1, especially _L_R113. In space, _L_R113 located at 3 Å away from the acidic amino acid _N_D99, favoring to enhance the alkalinity of _L_R113. In fact, the special coulomb interaction can reduce the dissociation degree of Nano/PD-L1. Unexpectedly, the p*K*_a_ value of _L_Y123 reduced from 16.09 to 11.07. In PD-1/PD-L1, _L_Y123 was embedded in PD-1 groove containing E136 [12] and interacted with Y68 through a strong π–π stacking effect [51], while there were more hydrophobic amino acids around _L_Y123 (such as _L_A121, _N_A114, _N_F101, _N_Y117 and _L_M115) in Nano/PD-L1. The alteration of pocket environment led to the p*K*_a_ values change of _L_Y56, _L_R113, _L_Y123 and _L_R125, which promotes the molecular recognition of PD-L1 by nanobody.

### 2.6. Molecular Recognition

Conformational change mentioned above and molecular recognition are two important scientific issues in the process of drug discovery. The design and screening of lead compounds based on PD-1/PD-L1 complex is a challenge due to the active site just being a protein-protein interaction (PPI) pattern. An adequate understanding PPI surface active sites and its intermolecular interactions will help to mitigate the pessimistic situation of discovering anti-PD-1/PD-L1 lead compounds.

**Figure 11:**
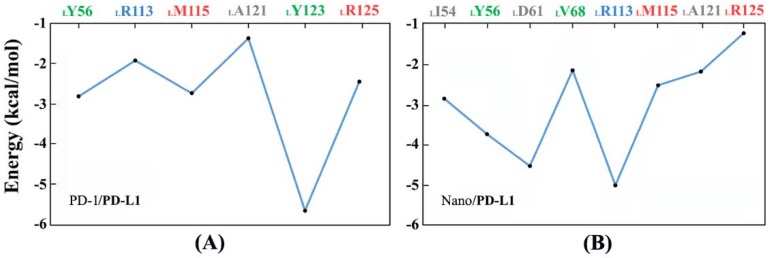
The key residues of PD-L1 binding with PD-1 (**A**) and nanobody (**B**) acquired by the Molecular Mechanics/Generalized Born Surface Area (MM/GBSA)method.

First of all, Molecular Mechanics/Generalized Born Surface Area (MM/GBSA)energy decomposition was utilized to predict the key residues favoring the formation of the PD-1/PD-L1 and Nano/PD-L1 systems. Figure 11 shows the pivotal residues with binding energies less than −1 kcal·mol^−1^. From Figure 11A, it is found that _L_Y56, _L_R113, _L_M115, _L_A121, _L_Y123 and _L_R125 residues all have relatively lower binding energy in the PD-1/PD-L1 system and _L_Y123 may be particularly important for PD-1/PD-L1 recognition after jointly considering Figure 10. Figure 11B shows eight key residues (i.e., _L_I54, _L_Y56, _L_D61, _L_V68, _L_R113, _L_M115, _L_A121 and _L_R125) in Nano/PD-L1 with energies less than −1 kcal·mol^−1^, two more residues than that of PD-1/PD-L1. Moreover, the binding energy of _L_R113 significantly decreased from −1.92 to −5.02 kcal·mol^−1^ by MM/GBSA method, thus _L_R113 is identified as one of the key residues in the recognition of antibodies and receptors, which is consistent with the previous analyses of dihedral distribution (see Figure S6) and p*K*_a_ (see Figure 10).

**Figure 12:**
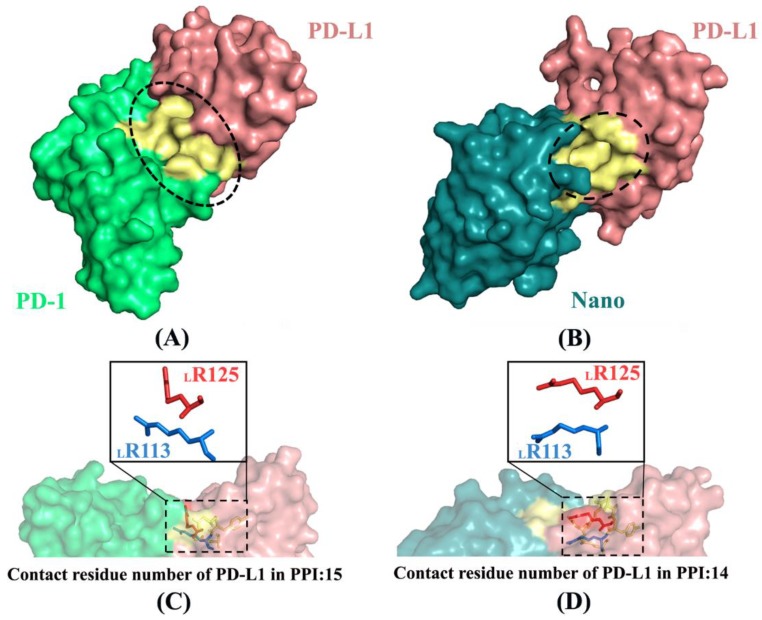
Contact surfaces (**A**,**B**) and conformational changes of two key residues (**C**,**D**) in the PD-1/PD-L1 (**A**,**C**) and Nano/PD-L1 (**B**,**D**) systems. PD-1 is shown in green and PD-L1 is in pinkish brown and the yellow portion primarily expresses the surface composed of _L_R113 and its surrounding amino acid residues. The ovals represent the shape of the interface between protein and protein and the boxes are residues with a large change in conformation.

PD-L1 binds to PD-1 or Nano with multiple non-bonded interactions between amino acids distributing on PPI such as hydrogen bonds, salt bridges and hydrophobic interactions [12,30] (more details see Figure S7). Figure 12 shows the contact surfaces and conformational changes of two key residues (i.e., _L_R113 and _L_R125) in the PD-1/PD-L1 and Nano/PD-L1 systems. There are three small and deep grooves in the PPI of PD-1/PD-L1 (see Figure 12A), while just only one deep binding groove in the same position of Nano/PD-L1 (see Figure 12B), confirming that the antibody binds to PD-L1 admirably. In addition, similar to the literature data [12,30], there are 15 and 14 residues of PD-L1 involved in the formation of PPI for the PD-1/PD-L1 and Nano/PD-L1 systems, respectively. After the association of PD-1 and nanobody, the conformations of _L_R113 and _L_R125 changed significantly (see Figure 12C,D). In detail, _L_R113 regulated the position of terminal amino group in order to better interact with the opposite _N_D99 in the Nano/PD-L1 system and _L_R125 approaches to _L_R113 so that the conformation of _L_R125 was also adjusted to accommodate _L_R113.

Figure S8 shows the dihedral angle variations of _L_R113 (in blue) and _L_R125 (in red) over simulation time. The radius of watch face in the figure is used to represent the simulation time (total 100 ns). The dihedral angle (Φ) of _L_R113 in PD-1/PD-L1 fluctuates between 200° and 290° but ranged from 190° to 270° in Nano/PD-L1. In addition, the fluctuation of the dihedral angle (Ψ) of _L_R125 (see Figure S8C,D) in the two systems is 120°–180° and 110°–165°, respectively. The decrease of the angle range proves that _L_R113 and _L_R125 both play a key role in the conformational change and flexibility reduction of β-sheet groups. In conclusion, the binding of nanobody distorts PD-L1’s conformation and reduces the flexibility of the recognition region, especially in the β-sheet groups region.

Salt bridge plays an important role in maintaining protein stability and participating in protein biological functions, which is divided into half salt-bridge, single salt-bridge and bidentate salt-bridge [52,53]. Generally speaking, when the distance between the O atom of the Asp/Glu side chain and the N atom of the Arg/Lys/His side chain is less than 4 Å, the salt bridge can be formed [54]. In PD-1/PD-L1, total 6 strong intramolecular salt bridges (i.e., R94-NH1/D117-OD1, R94-NH2/D117-OD2, _L_Q91-NE2/_L_D90-OD1, S55-OG/D105-OD2, _L_R84-NH1/_L_D108-OD2 and _L_R84-NH2/_L_D108-OD1) were formed. In Nano/PD-L1, however, we observed 7 almost different salt bridges, including _N_R67-NH1/_N_D90-OD2, _N_R67-NH2/_N_D90-OD1, _N_R38-NH1/_N_D90-OD1, _N_T107-OG1/_N_D103-OD2, _L_R84-NH1/_L_D108-OD2, _N_R45-NE/_N_Q39-OE1 and _L_R84-NH1/_L_D108-OD1. Compared with PD-1/PD-L1, the number of intermolecular hydrogen bonds also increased from 1 (i.e., Y68-OH/ _L_D122-OD1) to 3 (i.e., _L_R113-NH2/_N_D99-OD2, _L_R113-NH1/_N_D99-OD2 and _L_R125-NH2/_N_Q116-OE1) in Nano/PD-L1, which may lead to the obvious conformational change of _L_R113 and _L_R125. We subsequently calculated the distances of _L_R113-NH1/2 with _N_D99-OD2 and of _L_R125-NH2 with _N_Q116-OE1 in Nano/PD-L1 (see Figure S9), according to geometric criteria, finding that all salt bridges remained stable in the simulation process. Presumably, antibodies might damage the association of PD-1 with PD-L1 by strengthening salt bridge network.

Hydrogen bonds have specificity and closely relate to protein function comparing with other interactions (such as ionic interactions, hydrophobic interactions), which plays a crucial role in maintaining structural stability and identifying ligands. To subtly reveal the hydrogen bonds interactions of PD-L1 with nanobody and PD-1, the detailed analyses of intermolecular hydrogen bonds with occupancy over 40% were performed in the PD-1/PD-L1 and Nano/PD-L1 systems. The hydrogen bond is determined by the geometric criterion [55], that is, the angle of acceptor-hydrogen-donor is greater than 135° and the distance between acceptor and hydrogen atoms is less than 3.5 Å. It is found that there is no intermolecular hydrogen bond in PD-1/PD-L1, while a novel hydrogen bond (_N_T105-OG1-HG1/_L_Q66-OE1) is formed between Nano and PD-L1. Fewer hydrogen bond differences may only specially, rather than enthalpically, contribute to molecular recognition. Thus, we speculate that hydrophobic interactions may play more important role for the affinity than hydrogen bonds, especially with surface binding. Therefore, we calculated the solvent accessible surface area (SASA) and the buried areas of relevant systems.

SASA is one of the important parameters for biomolecular recognition. From Figure S10A/C, it can be seen that the SASAs of five systems did not change significantly during MD simulations. The total mean buried area (654 ± 46 Å^2^) of Nano/PD-L1 is distinctly smaller than that of PD-1/PD-L1 (932 ± 54 Å^2^), indicating that PD-L1 is buried more fully than nanobody within PD-1. In fact, the nonpolar interactions could be more conductive to the association of PD-L1 with PD-1.

In conclusion, the PPI of PD-1/PD-L1 is different from Nano/PD-L1, such as the number of salt bridge changing from 1 to 3, which of intermolecular hydrogen bonds increasing from 0 to 1. These suggest that the polar interactions including salt bridge, hydrogen bond and solvent effect cause PD-L1 to bind nanobody more tightly and selectively than to bind PD-1. It is speculated that all of the above favorable polar interactions may obviously offset the unfavorable nonpolar interactions. To investigate more quantitative protein-protein recognition, the binding free energy calculation of PD-L1 with nanobody and PD-1 was performed later.

### 2.7. Binding Free Energy Calculation

The binding free energy can effectively evaluate molecular recognition between receptor and ligand, characterizing the strong or weak binding capacity such as the inhibitory capacity in the physiological activity and so forth. This work predicts the binding free energies of the PD-1/PD-L1 with Nano/PD-L1 systems using MM/PBSA method. Table 3 shows that the total enthalpy change and entropy variety of Nano/PD-L1 are −56.73 and −42.83 kcal·mol^−1^, respectively. Then, the calculated absolute binding free energy (∆*G*_bind_) is −13.90 kcal·mol^−1^, which is well consistent with the reported experimental value (−11.38 kcal·mol^−1^). In contrast to PD-1/PD-L1 with the ∆*G*_bind_ of −5.27 kcal·mol^−1^, nanobody and PD-L1 have lower binding free energy values, thus the destruction of endogenous ligand-receptor binding is beneficial to T cells proliferation and effectively inhibits tumor cells growth.

The free energy can be decomposed into polar (*E*_ELE_ + *G*_PBELE_) and nonpolar (*E*_VDW_ + *G*_PBSUR_) parts according to the MM/PBSA method. In detail, the four core values (i.e., *E*_ELE_, *G*_PBELE_, *E*_VDW_, *G*_PBSUR_) in PD-1/PD-L1 and Nano/PD-L1 are −284.33/323.56/−73.06/−12.81 and −317.78/339.76/−70.00/−8.71 kcal·mol^−1^, respectively. It can be found that the nonpolar interactions can promote the formation of PD-1/PD-L1 and Nano/PD-L1 complexes, being the important driving force for their binding. In terms of the electrostatic part, the positive sum of *E*_ELE_ and *G*_PBELE_ indicates that the polar interactions are unfavorable for molecular recognition. Nevertheless, the association of PD-L1 with nanobody has greatly weakened the adverse trend than that with PD-1 by strengthening the interactions of salt bridge, which may be one of the main factors to maintain relatively lower binding energy.

### 2.8. The Role of Solvents in Molecular Recognition

PPIs controls many biological processes, such as cell proliferation, growth, differentiation, signal transduction and apoptosis, which has become an important drug target for novel human immunization therapy [56]. Protein must be in the solvent water environment to fully play its physiological role, so the solvation effect is also an important part of molecular recognition studies. The pocket between PD-1 and PD-L1 is shallow enough to hold water molecules that penetrates the interface between protein-protein to aid the recognition of PD-1 by PD-L1.

**Figure 13:**
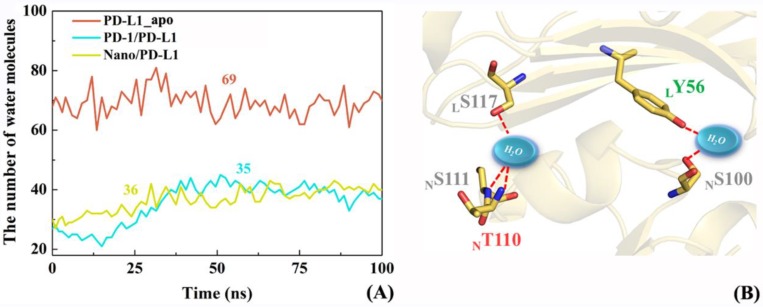
The change of number of water molecules at PD-1_apo, PD-1/PD-L1 and Nano/PD-L1 interfaces with time (**A**); and water-medicated hydrogen bonds in the Nano/PD-L1 system (**B**).

The changes of the number of interface water in PD-L1_apo, PD-1/PD-L1 and Nano/PD-L1 systems over time were briefly examined to investigate whether interfacial water molecules mediate some intermolecular hydrogen bonds. Based on the snapshots sampled every 500 ps for 100 ns, Figure 13A shows the alteration of the number of water molecules within 4 Å around the β-sheet groups for the PD-L1_apo, PD-1/PD-L1, Nano/PD-L1 systems. The average numbers of water molecules are 69, 35 and 36 in the three systems, implying the similar solvation environment of the PPIs in the two complex systems. In addition, the number of PD-1/PD-L1 interface water increased slightly from 0 to 50 ns, reflecting the expansion of the PD-L1/PD-L1 and Nano/PD-L1 systems during the MD simulation process, which is consistent with the previous contact residue analyses (see Figure 7). In the Nano/PD-L1 system, two interface water molecules exist most of the time, forms the stable water-mediated hydrogen bonds with _L_S117, _N_S111, _N_T110, _L_Y56 and _N_S100 in turn.

**Figure 14:**
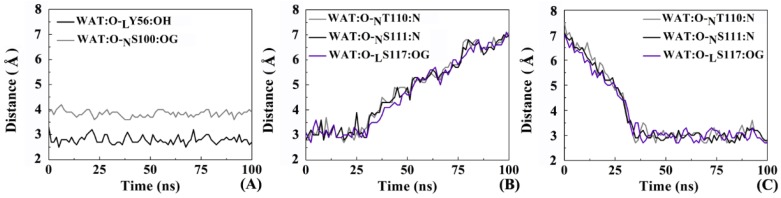
The distance between the hydrogen bond acceptor and donor varies with time. (**A**) _L_Y56 (black line) and _N_S100 (gray line) with water molecule; (**B**) _N_T110 (gray line), _N_S111 (black line) and _L_S117 (purple line) with water molecule; (**C**) _N_T110 (gray line), _N_S111 (black line) and _L_S117 (purple line) with water molecule.

Observing the trajectories of MD simulations, it was found that the interface water between the antibody and the receptor shifts orderly. Figure 14 shows the distance of the hydrogen bonds involved in several representative interface water molecules versus time. Two interesting features can be concluded. (1) One water molecule always preserves a steady hydrogen bond between _L_Y56 and _N_S100; (2) Most of hydrogen bonds exist throughout the MD process, nevertheless, the participating water molecules are dynamic. For example, two water molecules both are involved in the formation of hydrogen bonds with _N_T110, _N_S111 and _L_S117. To sum up, the interfacial water molecules change dynamically but the total quantity is almost constant. Solvation effect plays an important role in maintaining the recognition between nanobody and PD-L1 through several stable hydrogen bonds, helping the antibody to exert its inhibition activity more effectively.

### 2.9. Inhibition Mechanism and Implications for Drug Design

**Figure 15:**
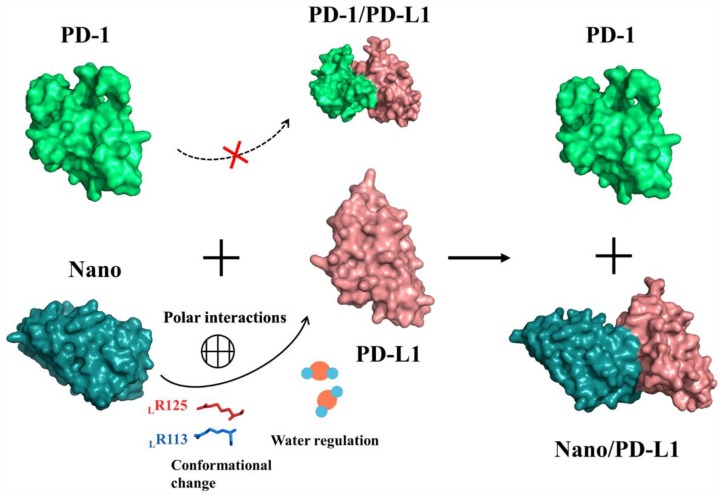
The possible inhibition mechanism of monoclonal antibody.

The characteristic of Immune checkpoint molecules including PD-1/PD-L1 and monoclonal antibodies for immunotherapy both are widely studied. T-cells and tumor cells both are well recognized by PD-1/PD-L1 molecules on their surface and the proliferation of T lymphocytes and the release of cytokines were restrained, leading to the phenomenon of immune escape of tumor cells [44]. According to the analyses of RMSD and time-dependent RMSF, the contact of PD-1/PD-L1 is not stable and the whole system exhibits a slight tendency of enlargement. The free energy calculation and energy decomposition results (Table 3 and Figure 11) contribute to the speculation on the possible inhibition mechanism of nanobody. The nanobody, with stronger binding ability to the PD-L1 PPI surface than that of PD-1, can competitively suppress the formation of PD-1/PD-L1 complex through the regulation of polar interactions, conformational change and solvent effect and so forth. (see Figure 15). Moreover, the consequence of structure stacks (see Figure 6) and residue dihedral distribution (see Figure S6) both reveal that the antibody alters the local conformation of PD-L1. The main backbone of _L_R113 and _L_R125 in F region rotates and forms a steady polar interaction with nanobody (_N_D99 and _N_Q116), reducing the flexibility of Nano/PD-L1. The analyses of free energy landscape, clustering and energy decomposition all indicate that the strong binding potential reduces the motion range of the Nano/PD-L1 system and a more stable conformational state is obtained, which will benefit antibodies to exert its inhibitory activity.

Study on the process of drug antibody development and PD-L1 receptor structural biology shows that the FG area [11,20,33] is vital for drug design and remodeling. Meanwhile, our molecular simulation results indicate that _L_Y56, _L_R1113, _L_RM115, _L_A121 and _L_R125 located in the FG region all are the key residues for the recognition of PD-L1 by nanobody. Energy decomposition indicates that _L_R113 forms salt bridges and hydrogen bonds with _N_D99. A hydrogen bond is formed between _L_R125 and _N_Q116 but the stability is slightly lower. If _N_Q116 is mutated to acidic amino acids such as aspartic acid or glutamic acid, with the help of salt bridge, the binding capacity of _L_R113 with nanobody will be greatly improved. To sum up, in the optimization of antibody, focusing on _L_R113 and _L_R125, mutations of these residues may help to improve the identification of antibodies and the PD-L1 β region groups, thus reduce the IC_50_ value of nanobody. 

## 3. Materials and Methods

All computational studies were carried out in four single PCs, running on two Intel Xeon E5-2643v3 processors with 32 GB RAM and 2 TB hard disk with Red Hat Linux Enterprise version 6.5 (Red Hat, Inc., Raleigh, NC, USA) as the operating system.

### 3.1. Molecular Dynamics Simulations

There were five systems for MD simulation which were PD-1 monomer, PD-L1 monomer, PD-1/PD-L1, nanobody and nanobody/PD-L1 complexes in this work, named by PD-1_apo, PD-L1_apo, Nano, PD-1/PD-L1 and Nano/PD-L1, respectively. PD-1_apo was the extracellular IgV of PD-1 (PDB code: 3RRQ) and PD-L1_apo structure was adopted in the extracellular IgV of PD-L1 (PDB code: 5C3T). PD-1/PD-L1 was obtained from the extracellular IgV of PD-1/IgV complexed with PD-L1 (PDB code: 4ZQK). Nano was from Fc fragment in human IgG1 (PDB code: 5JDS). Nano/PD-L1 was acquired from the lgV of PD-L1/Fc fragment in human IgG1 (PDB code: 5JDS). 

All MD simulations were performed using AMBER 12 software package [57] and AMBER force field [58]. The simulation temperature was set at 300 K and the TIP3P water model was applied [59]. total 6618/6351/14015/6202/14776 water molecules and 1 Cl^−^/1 Na^+^/0 Cl^−^/4 Cl^−^/3 Cl^−^ countra-ions were added to the PD-1_apo, PD-L1_apo, PD-1/PD-L1, Nano and Nano/PD-L1 systems, respectively, with the box boundary of 10 Å. The five systems were optimized twice subsequently. The first is that the systems were optimized when the solute is constrained (force constant is 2.09 × 10^5^ kJ·mol^−1^·nm^−2^), consisting of the steepest descent of 5000 steps and the conjugate gradient procedure of 5000 steps; The second optimization was in the cased of complete removal of the constraints with the steepest descent of 5000 steps and the conjugate gradient of 5000 steps. The energy convergence threshold of the two minimizations was less than 4.182 × 10^−4^ kJ·mol^−1^·nm^−2^. After the energy minimization was completed, the productive MD simulations were started, which were also divided into two procedures. First of all, solute was constrained with the force constant of 41.82 kJ·mol^−1^·nm^−2^ and the temperature gradually increased from 0 to 300 K in the initial 2.5 ns; Afterwards, a 97.5 ns unconstrained MD simulation was performed adopting SHAKE algorithm [60] to constrain the hydrogen-containing atoms with non-bonded interaction radius of 10 Å. The integration step was set as 2 fs and the conformation was collected every 1 ps, thus total 100,000 conformations were collected for the following structural analyses.

### 3.2. Free Energy Landscape

The main idea of free energy landscape (FEL) is considering the minimum value of free energy surface (corresponding to the most stable state of the system) and demarcation point (relating to a transient state in the process of system change) to investigate molecular movement and conformational changes for biology systems. In addition, principal component analysis (PCA) is widely used to describe the most important kinetic processes in the system. The first principal component (PC1) and the second principal component (PC2) both serve as reaction coordinates for the mapping of free energy surface diagram [61]. Free energy is defined as:

(1) $$\Delta G\left(X\right)=-k_{B}T\ln P\left(X\right)$$

Here, the reaction coordinate X is PC1, 2 and *k*_B_ is the Boltzmann constant. *T* expresses the absolute temperature in Kelvin. *P*(X) represents the contribution of a particular conformation to the overall PCs, apprehended as the probability of conformational distribution. In this work, PC1 and PC2 calculations were the basis for FEL and subsequent cluster analyses to investigate conformational changes in systems.

### 3.3. Cluster Analysis

Cluster analysis of 100,000 molecular conformations obtained from MD simulations in the PD-1/PD-L1 and Nano/PD-L1 systems was performed using the MMTSB software package [49]. The basic idea of cluster analysis is calculating the root mean square deviation (RMSD) values of C_α_ between the various conformations gradually and establishing the RMSD matrix (N × N where N is the number of conformations). Assuming a RMSD threshold, if the RMSD between two arbitrary conformations smaller than this threshold, they are grouped into one certain cluster, in which the lowest energy conformation is taken as the representative conformation. The cluster formula used is as follows. When ΔRMSD is less than the threshold of 1.7 Å, *C* = 1 indicates that the two conformations are in the same cluster. Conversely, *C* = 0 indicates that they are not in the same cluster.

(2) $$C=\{\begin{array}{ccc} 1 & for & \Delta \mathrm{RMSD}\leq 0.17\\ 0 & for & \Delta \mathrm{RMSD}>0.17 \end{array}$$

Moreover, the computational formula of RMSD is:

(3) $$\mathrm{RMSD}=\sqrt{\frac{1}{N}\sum_{i=1}^{N}\delta_{i}^{2}}$$

where *N* is the total number of atoms, while *δ* is the translational distance between corresponding atoms in two different conformations.

### 3.4. Solvent Accessible Surface Area (SASA) Calculation

SASA is one of the key parameters to be considered in biomolecular recognition research [62,63]. SASA is generally defined as the surface area formed by a water probe ball rolling around the surface of biomolecules. The larger the SASA, the stronger the hydrophobic effect. In the actual calculation, SASA can be divided into protein residual level. It is worth mentioning that some amino acids are buried in the proteins deeply and the corresponding SASA is zero. In addition, buried SASA (*S*_buried_) is an important criterion for evaluating the strength and effectiveness of molecular recognition between proteins. The computational formula is as follows:

(4) $$S_{\mathrm{buried}}=\left(S_{A}+S_{B}-S_{\mathrm{AB}}\right)/2.0$$

Here, *S*_buried_ stands for the buried SASA, while *S_A_*, *S_B_* and *S_AB_* are used to describe the SASAs of protein A, protein B and complexes AB, respectively. In this work, the Gromacs soft package [64] was used to calculate the SASAs of PD-1_apo (*S*_PD-1_), PD-L1_apo (*S*_PD-L1_), Nano (*S*_nano_), PD-1/PD-L1 (*S*_PD-1/PD-L1_) and Nano/PD-L1 (*S*_nano/PD-L1_) systems, as well as the buried areas (*S*_buried_) of both complexes.

### 3.5. Binding Energy Predication

The conformations were extracted from the MD trajectories of the PD-1/PD-L1 and Nano/PD-L1 complex systems every 0.5 ns intervals from 1 to 100 ns. The average binding free energy based on the total 200 conformations was calculated with the method of MM/PBSA. The formula is as:

(5) $$\Delta G_{\mathrm{bind}}=\Delta H-T\Delta S=(\Delta E_{\mathrm{VDW}}+\Delta E_{\mathrm{ELE}}+\Delta G_{\mathrm{PBELE}}+\Delta G_{\mathrm{PBSUR}})-T\Delta S$$

where Δ*E*_VDW_ refers to the non-polar fraction of the intramolecular energy under vacuum, while Δ*E*_ELE_ indicates the electrostatic section. Δ*G*_PBELE_ and Δ*G*_PBSUR_ correspond to the hydrophilic and hydrophobic parts of the solvation binding free energy, respectively. Δ*H* represents the total enthalpy change and *T* is the absolute temperature in Kelvin. Δ*S* refers to the total entropy change calculated using the normal mode method [65].

### 3.6. Key Residue Scanning

MM/GBSA (molecular mechanics/generalized born surface area) energy decomposition [66,67] method was used to search key residues of receptor-ligand recognition. Specifically, the MM/GBSA method divides the binding energy of each residue into the vacuum intramolecular energy calculated by molecular mechanics method, the polar solvation energies calculated by the generalized Born (GB) model [68,69] and the nonpolar solvation energies computed by linear combinations of pairwise overlaps (LCPO) model [70,71]. The LCPO algorithm defines that the non-polar solvation energy has a high positive correlation with the solvent accessible surface area (SASA). Afterwards, the binding energy of each residues was also decomposed into the main and side chain atoms.

## 4. Conclusions

Four 100 ns MD simulations were performed for the PD-1_apo, PD-L1_apo, Nano, PD-1/PD-L1 and Nano/PD-L1 systems, after structural complementation and verification of their rationality. The good correlation between calculated and the experimental B-factor values, stable potential energy and small-scale fluctuations of RMSD, all prove the reliability of the simulation results. Besides, the relatively high RMSD of PD-1/PD-L1 demonstrates the short half-life in its natural state and low affinity of PD-1 with PD-L1, meanwhile, the lower RMSD of the Nano/PD-L1 indicating that the nanobody binds PD-L1 strongly and forms a stable complex. The RMSF value of C_α_ atoms in the PD-1/PD-L1 system exhibits a higher flexibility than that in Nano/PD-L1 due to the fact that Nano restricts the motion amplitude of PD-L1, especially the β-sheet groups, which is consistent with the time-dependent RMSF analysis and more residual contacts between Nano and PD-L1 also support lower flexibility of Nano/PD-L1.

The analyses of FEL and conformational clustering also indicate that the Nano/PD-L1 had a smaller conformational transition space than PD-1/PD-L1. The movement direction and amplitude of PD-L1 promotes its association with antibody. In conclusion, That CC’FG remains low flexibility and conformational variety and transition space become slight narrower, may be one of the pivotal reasons for the high activity of antibodies.

In terms of molecular recognition, energy decomposition implies that the binding site of antibody with PD-L1 are mainly located at the interface of PD-1/PD-L1 and _L_R113 is the most vital residue for the association. Compared with the endogenous PD-1/PD-L1 complex, Nano/PD-L1 has stronger polar interactions including more intramolecular, intermolecular salt bridges. Furthermore, slight difference of hydrogen bonds may aid the specific identification of PD-L1 by nanobody. The statistical analysis of interface water reveals that the water-mediated hydrogen bond makes great contribution to the recognition between ligands and PD-L1 receptor. These important polar interactions help the identification of nanobody and PD-L1, help nanobody to exert its inhibitor activity and also contribute to the subsequent anticancer drug design and modification.

The contribution of _L_R113 and _L_R125 to molecular identification is highlighted by the analyses of both two-dimensional distribution of dihedral angles and p*K*_a_ values (13.89 → 15.92 and 11.55 → 12.13, respectively) calculation. Then, observing the three-dimensional structures of _L_R113 and _L_R125, exploring inhibitory mechanism of nanobody, both provide possible guidance for the modification of antibodies, such as mutating _N_D99 and _N_Q116 into acidic amino acids, which may help to increase the binding capacity between nanobody and PD-L1. In sum, this work is based on a deep understanding of the molecular recognition and inhibition mechanisms of nanobody and finally enhances the efficiency of antibody modification.

## Figures and Tables

**Table 1:** Bioinformatics data for PD-1/PD-L1 structures currently available.

Classification	PDB Codes	Residue Ranges	Substrates	Features
PD-1_apo	1NPU, 3RRQ, 2M2D	S1-E150	none	All are monomers with fewer mutation. 3RRQ comes from the human with the resolution of 2.1 Å [24,25].
PD-1_antibody	5GGR, 5GGS, 5JXE, 5B8C, 5WT9	L25-E146	Nivolumab, Pembrolizumab	All structures belong to the human and are intact with high-resolution, which can be used to explore the binding mode of PD-1 with antibody inhibitors [18,19,26,27].
PD-L1_apo	3BIS, 3FN3, 4ZI8, 5JDR, 5C3T	M18-A232	none	All are dimers with resolutions up to 1.8 Å (such as 5CT3). Since there is no substrate information, it is used to investigate conformational changes before binding its inhibitors [12,28,29,30].
PD-L1_antibody	5GGT, 5GRJ, 5JDS, 5X8L, 5X8M	A18-H233	BMS-936559, avelumab, KN035-Fc, atezolizumab, durvalumab	5JDS has the complete structure, with no mutation and a high resolution of 1.7 Å. The substrate in 5JDS is a nanobody, being the latest antibody drugs which provides a useful idea for the reconstruction and design of antibodies based on the structure of PD-L1 [19,30,31,32].
PD-1/PD-L1	3BIK, 3SBW, 4ZQK, 5IUS	D29-I148; A18-L231	none	3BIK and 3SBW are mouse PD-1/human PD-L1 complex. 4ZQK is human-derived wild type with high resolution and no mutation, more suitable for the following simulation [12,28,33].
PD-L1/PD-L1_ligand	5J8O, 5J89 5N2D, 5N2F	A18-H142	BMS-8, BMS-37, BMS-200, BMS-202	For the first time, the structural information of small molecule inhibitors of PD-L1 was reported. Based on the 5J89 structure, Zak group proposed a possible inhibitory mechanism of PD-L1 small molecule inhibitors [34,35].

**Table 2:** Research progress of antibodies targeting PD-1/PD-L1.

Targets	Antibodies	Developers	Property
PD-1	Nivolumab	Bristol-Myers Squibb	Most of the antibody structures are mainly based on PD-L2 and IgG4. The side effects main lie in destabilizing the complex structure of PD-1/PD-L2. Currently, Nivolumab and Pembrolizumab both were used to treat NSCLC and lymphoma.
	Pembrolizumab	Merck	
	AMP-224/AMP-514	GlaxoSmithKline	
	Pidilizumab	CureTech	
	BGB-A317	BeiGene	
	SHR-1210	Jiangsu Hengui Medicine	
PD-L1	BMS-936559	Bristol-Myers Squibb	PD-L1 antibody mainly comes from IgG1 and its side effect is weaker than PD-1 mAbs, which is mainly used for the treatment of advanced solid tumors. Most mAbs are in phase I clinical stage, in which Atezolizumab is approved for clinical use.
	Atezolizumab	Genentech/Roche	
	Durvalumab	AstraZenerca	
	Avelumab	Pfizer/Merck	
	KN035-Fc	Hongqiao I Ins Med	
PD-L2	rHlgM12B7	Mayo Clinic/NCI	The effect of PD-L2 on T cells is far less than that of PD-L1. There are few reports on mABs of PD-L2.

**Table 3:** The predications of binding free energy in the PD-1/PD-L1 and Nano/PD-L1 systems (kcal·mol^−1^).

Systems	∆*H*	*T*∆*S*	∆*G*_bind_	∆*G*_exp_
PD-1/PD-L1	−46.64 ± 3.24	−41.37 ± 1.49	−5.27	−
Nano/PD-L1	−56.73 ± 5.42	−42.83 ± 2.68	−13.90	−11.38 [30]

## Supplementary Materials

Supplementary materials can be found at http://www.mdpi.com/1422-0067/19/7/1984/s1.

## References

[1] Center M.M. (2015). Global cancer statistics. CA-Cancer J. Clin..

[2] Stewart B., Wild C. (2014). World Cancer Report 2014.

[3] Eilard M.S., Lundgren L., Cahlin C., Strandell A., Svanberg T., Sandström P. (2017). Surgical treatment for gallbladder cancer—A systematic literature review. Scand. J. Gastroenterol..

[4] Feain I.J., Court L., Palta J.R., Beddar S., Keall P. (2017). Innovations in radiotherapy technology. J. Clin. Oncol..

[5] Rajasekaran T., Ng Q.S., Tan D.S., Lim W.T., Ang M.K., Toh C.K. (2017). Metronomic chemotherapy: A relook at its basis and rationale. Cancer Lett..

[6] Chen D.S., Mellman I. (2013). Oncology meets immunology: The cancer-immunity cycle. Immunity.

[7] Couzin-Frankel J. (2013). Breakthrough of the year 2013. Cancer immunotherapy. Science.

[8] Phan T.G., Long G.V., Scolyer R.A. (2015). Multiple checkpoints on the long road towards cancer immunotherapy. Immunol. Cell Biol..

[9] Antonia S.J., Larkin J., Ascierto P.A. (2014). Immuno-oncology Combinations: A Review of Clinical Experience and Future Prospects. Clin. Cancer Res..

[10] Topalian S.L., Drake C.G., Pardoll D.M. (2015). Immune checkpoint blockade: A common denominator approach to cancer therapy. Cancer Cell..

[11] Hao G., Wesolowski J.S., Jiang X., Lauder S., Sood V.D. (2015). Epitope characterization of an anti-PD-L1 antibody using orthogonal approaches. J. Mol. Recognit..

[12] Zak K.M., Kitel R., Przetocka S., Golik P., Guzik K., Musielak B. (2015). Structure of the Complex of Human Programmed Death 1, PD-1 and Its Ligand PD-L1. Structure.

[13] Riella L.V., Paterson A.M., Sharpe A.H., Chandraker A. (2012). Role of the PD-1 Pathway in the Immune Response. Am. J. Transplant..

[14] Hawkes E.A., Grigg A., Chong G. (2015). Programmed cell death-1 inhibition in lymphoma. Lancet Oncol..

[15] Wherry E.J. (2011). T cell exhaustion. Nat. Immunol..

[16] Brahmer J.R., Tykodi S.S., Chow L.Q., Hwu W.J., Topalian S.L., Hwu P. (2012). Safety and Activity of Anti–PD-L1 Antibody in Patients with Advanced Cancer. New. Engl. J. Med..

[17] Zhan M.M., Hu X.Q., Liu X.X., Ruan B.F., Xu J., Liao C. (2016). From monoclonal antibodies to small molecules: The development of inhibitors targeting the PD-1/PD-L1 pathway. Drug Discov. Today.

[18] Na Z., Yeo SP., Bharath SR., Bowler M.W., Balıkçı E., Wang C.I. (2016). Structural basis for blocking PD-1-mediated immune suppression by therapeutic antibody pembrolizumab. Cell Res..

[19] Lee J.Y., Lee H.T., Shin W., Chae J., Choi J., Kim S.H. (2016). Structural basis of checkpoint blockade by monoclonal antibodies in cancer immunotherapy. Nat. Commun..

[20] Ibrahim R., Stewart R., Shalabi A. (2015). PD-L1 blockade for cancer treatment: MEDI4736. Semin. Oncol..

[21] Zak K.M., Grudnik P., Magiera K., Dömling A., Dubin G., Holak T.A. (2017). Structural Biology of the Immune Checkpoint Receptor PD-1 and Its Ligands PD-L1/PD-L2. Structure.

[22] Ahmed M., Barakat K.H. (2017). The Too Many Faces of PD-L1: A Comprehensive Conformational Analysis Study. Biochem.-US.

[23] Liu W., Liu G. Mapping Paratope and Epitope Residues of Antibody Pembrolizumab via Molecular Dynamics Simulation. Proceedings of the International Symposium on Bioinformatics Research and Applications.

[24] Zhang X., Schwartz J.C., Guo X., Bhatia S., Cao E., Lorenz M. (2004). Structural and functional analysis of the costimulatory receptor programmed death-1. Immunity.

[25] Cheng X., Veverka V., Radhakrishnan A., Waters L.C., Muskett F.W., Morgan S.H. (2013). Structure and Interactions of the Human Programmed Cell Death 1 Receptor. J. Biol. Chem..

[26] Horita S., Nomura Y., Sato Y., Shimamura T., Iwata S., Nomura N. (2016). High-resolution crystal structure of the therapeutic antibody pembrolizumab bound to the human PD-1. Sci. Rep.-UK.

[27] Tan S., Zhang H., Chai Y., Song H., Tong Z., Wang Q. (2017). An unexpected N-terminal loop in PD-1 dominates binding by nivolumab. Nat. Commun..

[28] Lin D.Y., Tanaka Y., Iwasaki M., Gittis A.G., Su H.P., Mikami B. (2008). The PD-1/PD-L1 complex resembles the antigen-binding Fv domains of antibodies and T cell receptors. Proc. Natl. Acad. Sci. USA.

[29] Chen Y., Liu P., Gao F., Cheng H., Qi J., Gao G.F. (2010). A dimeric structure of PD-L1: Functional units or evolutionary relics?. Protein Cell.

[30] Fei Z., Wei H., Wang X., Bai Y., Wang P., Wu J. (2017). Structural basis of a novel PD-L1 nanobody for immune checkpoint blockade. Cell Discov..

[31] Liu K., Tan S., Chai Y., Chen D., Song H., Zhang C.W. (2016). Structural basis of anti-PD-L1 monoclonal antibody avelumab for tumor therapy. Cell Res..

[32] Lee H.T., Lee J.Y., Lim H., Sang H.L., Yu J.M., Pyo H.J. (2017). Molecular mechanism of PD-1/PD-L1 blockade via anti-PD-L1 antibodies atezolizumab and durvalumab. Sci. Rep-UK.

[33] Pascolutti R., Sun X., Kao J., Maute R., Ring A.M., Bowman G.R. (2016). Structure and Dynamics of PD-L1 and an Ultra-High-Affinity PD-1 Receptor Mutant. Structure.

[34] Zak K.M., Grudnik P., Guzik K., Zieba B.J., Bogdan M., Alexander D. (2016). Structural basis for small molecule targeting of the programmed death ligand 1 (PD-L1). Oncotarget.

[35] Guzik K., Zak K.M., Grudnik P., Magiera K., Musielak B., Törner R. (2017). Small-Molecule Inhibitors of the Programmed Cell Death-1/Programmed Death-Ligand 1 (PD-1/PD-L1) Interaction via Transiently Induced Protein States and Dimerization of PD-L1. J. Med. Chem..

[36] Herbst R.S., Soria J.C., Kowanetz M. (2014). Predictive correlates of response to the anti-PD-L1 antibody MPDL3280A in cancer patients. Nature..

[37] Philips G.K., Atkins M. (2015). Therapeutic uses of anti-PD-1 and anti-PD-L1 antibodies. Int. Immunol..

[38] Naidoo J., Page D.B., Li B.T., Connell L.C., Schindler K., Lacouture M.E. (2015). Toxicities of the anti-PD-1 and anti-PD-L1 immune checkpoint antibodies. Ann. Oncol..

[39] Gunturi A., Mcdermott D.F. (2015). Nivolumab for the treatment of cancer. Expert Opin. Investig. Drugs.

[40] Gangadhar T.C., Salama A.K. (2015). Clinical applications of PD-1-based therapy: A focus on pembrolizumab (MK-3475) in the management of melanoma and other tumor types. Oncotargets Ther..

[41] Johnson D.B., Peng C., Sosman J.A. (2015). Nivolumab in melanoma: Latest evidence and clinical potential. Ther. Adv. Med. Oncol..

[42] Sharma P., Allison J.P. (2015). The future of immune checkpoint therapy. Science.

[43] Powles T., Eder J.P., Fine G.D., Braiteh F.S., Loriot Y., Cruz C. (2014). MPDL3280A (anti-PD-L1) treatment leads to clinical activity in metastatic bladder cancer. Nature..

[44] Alqudah D.A., Zihlif M.A., Taha M.O. (2016). Ligand-based modeling of diverse aryalkylamines yields new potent P-glycoprotein inhibitors. Eur. J. Med. Chem..

[45] Tomii K., Hirokawa T., Motono C. (2005). Protein structure prediction using a variety of profile libraries and 3D verification. Proteins.

[46] Feig M., Karanicolas J., Brooks C.L. (2004). MMTSB Tool Set: Enhanced sampling and multiscale modeling methods for applications in structural biology. J. Mol. Graph. Model..

[47] Hu J., Hu Z., Zhang Y., Gou X., Mu Y., Wang L. (2016). Metal binding mediated conformational change of XPA protein: A potential cytotoxic mechanism of nickel in the nucleotide excision repair. J. Mol. Model..

[48] Knapp B., Lederer N., Omasits U., Schreiner W. (2010). vmdICE: A plug-in for rapid evaluation of molecular dynamics simulations using VMD. J. Comput. Chem..

[49] Kabsch W., Sander C. (1983). Dictionary of protein secondary structure. Biopolymers.

[50] Dolinsky T.J., Nielsen J.E., Mccammon J.A., Baker N.A. (2004). PDB2PQR: An automated pipeline for the setup of Poisson-Boltzmann electrostatics calculations. Nucleic Acids Res..

[51] Ahmed M., Barakat K. (2017). When theory meets experiment: The PD-1 challenge. J. Mol. Model..

[52] Basu S., Mukharjee D. (2017). Salt-bridge networks within globular and disordered proteins: Characterizing trends for designable interactions. J. Mol. Model..

[53] Zhao W.S., Sun M.Y., Sun L.F., Liu Y., Yang Y., Huang L.D. (2016). A Highly Conserved Salt Bridge Stabilizes the Kinked Conformation of β2,3-Sheet Essential for Channel Function of P2X4 Receptors. J. Biol. Chem..

[54] Kumar S., Nussinov R. (1999). Salt bridge stability in monomeric proteins. J. Mol. Biol..

[55] Hu J.P., Gong X.Q., Su J.G., Chen W.Z., Wang C.X. (2008). Study on the molecular mechanism of inhibiting HIV-1 integrase by EBR28 peptide via molecular modeling approach. Biophys. Chem..

[56] Cummings C.G., Hamilton A.D. (2010). Disrupting protein-protein interactions with non-peptidic, small molecule alpha-helix mimetics. Curr. Opin. Chem. Biol..

[57] Case D.A., Darden T.A., Cheatham T.E., Simmerling C.L., Wang J., Duke R.E., Luo R., Walker R.C., Zhang W., Merz K.M. (2012). AMBER 12.

[58] Wang J., Cieplak P., Komllman P.A. (2000). How well does a restrained electrostatic potential (RESP) model perform in calculating conformational energies of organic and biological molecules. J. Comput. Chem..

[59] Jorgense W.L., Chandrasekhar J., Madura J.D., Impey R.W., Klein M.L. (1983). Comparison of simple potential functions for simulating liquid water. J. Chem. Phys..

[60] Ryckaert J.P., Ciccotti G., Berendsen H.J.C. (1977). Numerical integration of the cartesian equations of motion of a system with constraints: Molecular dynamics of n-alkanes. J. Chem. Phys..

[61] Hegger R., Altis A., Nguyen P.H., Stock G. (2007). How complex is the dynamics of Peptide folding?. Phys. Rev. Lett..

[62] Talavera D., Robertson D.L., Lovell S.C. (2011). Characterization of Protein-Protein Interaction Interfaces from a Single Species. PLoS ONE.

[63] Peri C., Morra G., Colombo G. (2016). Surface energetics and protein-protein interactions: Analysis and mechanistic implications. Sci. Rep..

[64] Kumari R., Kumar R., Lynn A. (2014). G_mmpbsa—A GROMACS tool for high-throughput MM-PBSA calculations. J. Chem. Inf. Model..

[65] Kottalam J., Case D.A. (1990). Langevin modes of macromolecules: Applications to crambin and DNA hexamers. Biopolymers.

[66] Kollman P.A., Massova I., Reyes C., Kuhn B., Huo S., Chong L. (2000). Calculating structures and free energies of complex molecules: Combining molecular mechanics and continuum models. Acc. Chem. Res..

[67] Catalysis E., Wang W., Donini O., Reyes C.M., Kollman P.A. (2001). Biomolecular simulations: Recent developments in force fields, simulations of enzyme catalysis, protein-ligand, protein-protein and protein-nucleic acid noncovalent interactions. Annu. Rev. Biophys. Biomol. Struct..

[68] Simonson T. (2001). Macromolecular electrostatics: Continuum models and their growing pains. Curr. Opin. Struc. Biol..

[69] Bashford D., Case D.A. (2000). Generalized born models of macromolecular solvation effects. Annu. Rev. Phys. Chem..

[70] Still W.C., Tempczyk A., Hawley R.C., Hendrickson T. (1990). Semianalytical treatment of solvation for molecular mechanics and dynamics. J. Am. Chem. Soc..

[71] Weiser J., Shenkin P.S., Still W.C. (1999). Approximate atomic surfaces from linear combinations of pairwise overlaps (LCPO). J. Comput. Chem..

