# Supplementary material for: PD-L1 Nanobody Competitively Inhibits the Formation of the PD-1/PD-L1 Complex: Comparative Molecular Dynamics Simulations
Source: Int J Mol Sci. 2018 Jul 7;19(7):1984. doi: 10.3390/ijms19071984 (PMC6073277; doi:10.3390/ijms19071984)
Supplement: Supplementary file 1 [file ijms-19-01984-s001.zip › supporting information/supporting information.docx]

PD-L1 nanobody competitively inhibits the formation of the PD-1/PD-L1 complex: Comparative molecular dynamics simulations

Xin Sun ^1,†^, Xiao Yan ^1,†^,Wei Zhuo ^2^, Jinke Gu ^2^, Ke Zuo ^1^, Wei Liu ^1^, Li Liang ^1^, Ya Gan ^1^,
Gang He ^1^, Hua Wan ^3^, Xiaojun Gou ^1^*, Hubing Shi ^4^, Jianping Hu ^1,^*

^1^ College of Pharmacy and Biological Engineering, Sichuan Industrial Institute of Antibiotics, Key Laboratory of Medicinal and Edible Plants Resources Development of Sichuan Education Department, Antibiotics Research and Re-evaluation Key Laboratory of Sichuan Province, Chengdu University, Chengdu610106, China; sunbsxw123@163.com(X.S.); yan_xiao163@163.com(X.Y); zuoke2015@outlook.com(K.Z.); liuwei@cdu.edu.cn(W.L.); lianglicdu@163.com(L.L.); Ganfcd@outlook.com(Y.G.); hegang@cdu.edu.cn(G.H.);

^2^ Ministry of Education Key Laboratory of Protein Science, Tsinghua-Peking Joint Center for Life Sciences, Beijing Advanced Innovation Center for Structural Biology, School of Life Sciences, Tsinghua University, Beijing, China; zhuowei1989@163.com(W.Z); gujinke@tsinghua.edu.cn(J.G)

^3^ College of Mathematics and Informatics, South China Agricultural University, Guangzhou, China; wanhua@scau.edu.cn

^4^ Laboratory of tumor targeted and immune therapy, Clinical Research Center for Breast, State Key Laboratory of Biotherapy, Sichuan University, and Collaborative Innovation Center for Biotherapy, Chengdu, China; shihubing77@sina.com

***** Correspondence: hjpcdu@163.com (J.H.); Tel.: +86-28-8461-6301; gxjmeprd@163.com (X.G.);

† These authors contributed equally to this work

Received: date; Accepted: date; Published: date


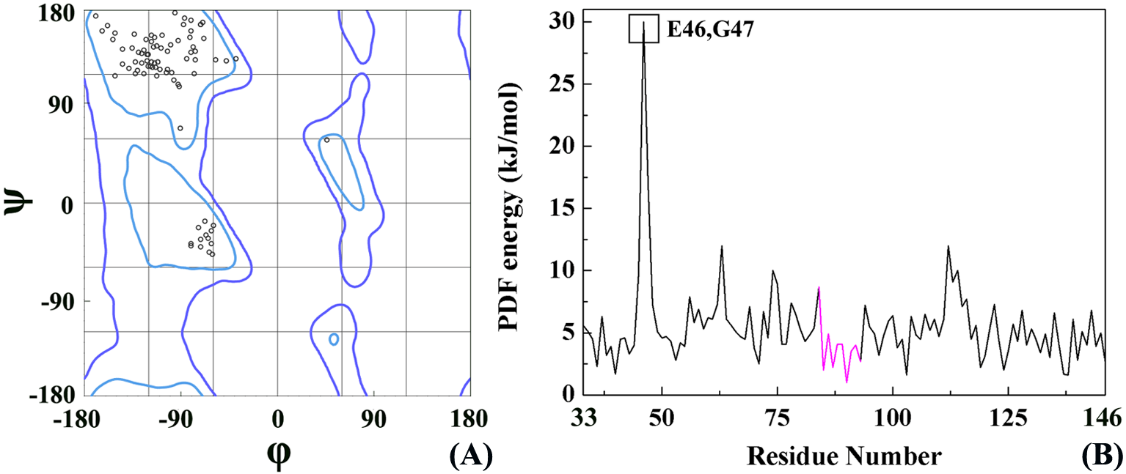


**Figure S1.** Ramachandran plot (**A**) and PDF energy (**B**) for each residue in PD-1. The unresolved residue section was marked in pink.
 Figure S2A shows the distributions of Root Mean Square Fluctuation (RMSF) of Cα atoms in PD-1/PD-L1 and PD-1_apo and PD-L1_apo .The flexibility distribution in the three systems trends to be similar, and the details suggest that three regions (LH69 ~ LL94, P39 ~ T51 and L128 ~ A132) of PD-1/ PD-L1 have higher RMSF values than those of PD-1_apo and PD-L1_apo, which is exactly identical with previous RMSD analyses and experimental literature [12] B-factor and RMSF both can be used to characterize conformational flexibility, and the larger B-factor and RMSF values indicate that the corresponding residues are more flexible. Figure S2B shows that the simulated B-factor values in PD-1/PD-L1 have high correlation with the experimental data (R2 = 0.40, N = 229), which suggests that the obtained MD trajectories are reliable and suitable for the subsequent analyses.


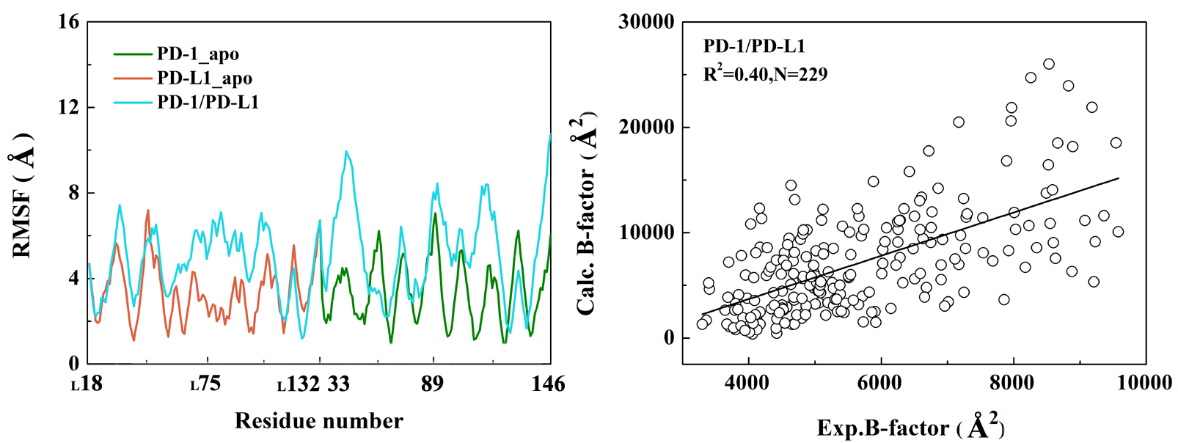


**Figure S2.** The comparative analyses of the trajectories for the three investigated systems. (**A**) RMSF distribution of C_α_ atoms. (**B**) The correlation between the calculated B-factor values and experimental data.


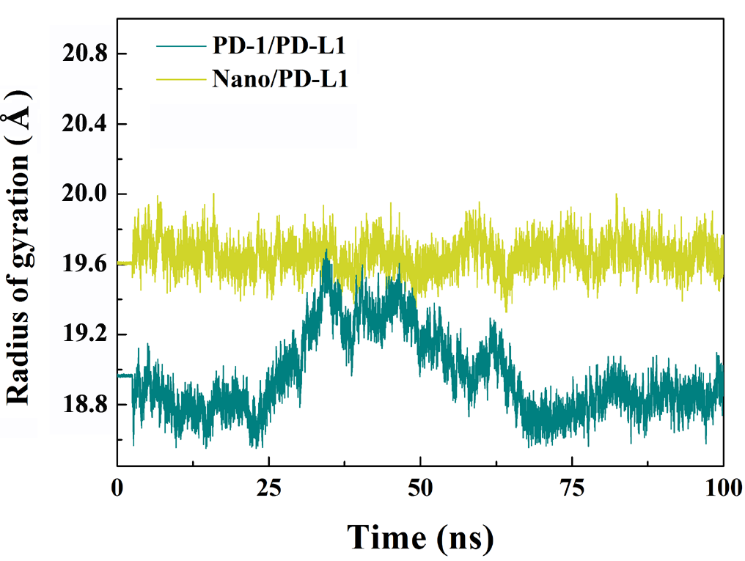


**Figure S3** The radius gyration of PD-1/PD-L1 and Nano/PD-L1.


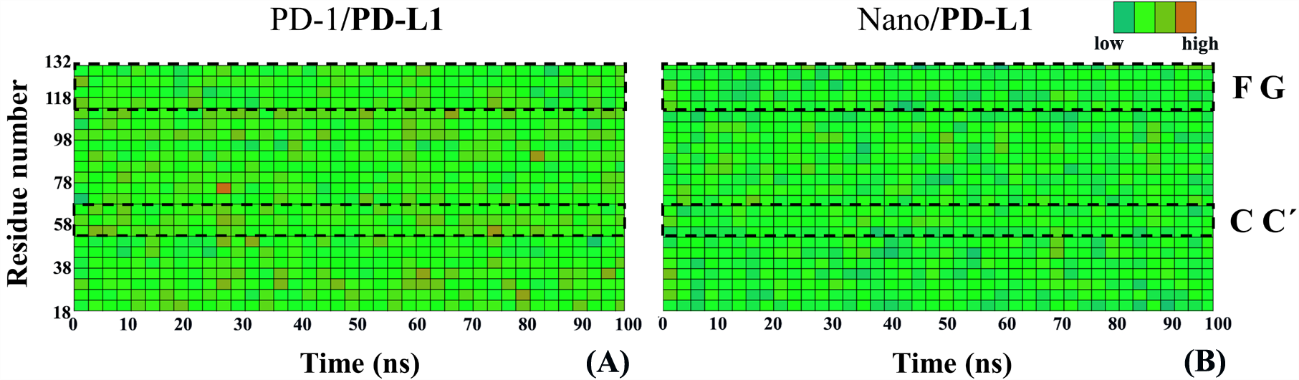


**Figure S4.** The RMSF change of C_α_ atoms of PD-L1 protein over time in the PD-1/PD-L1(**A**) and Nano/PD-L1 (**B**) systems.


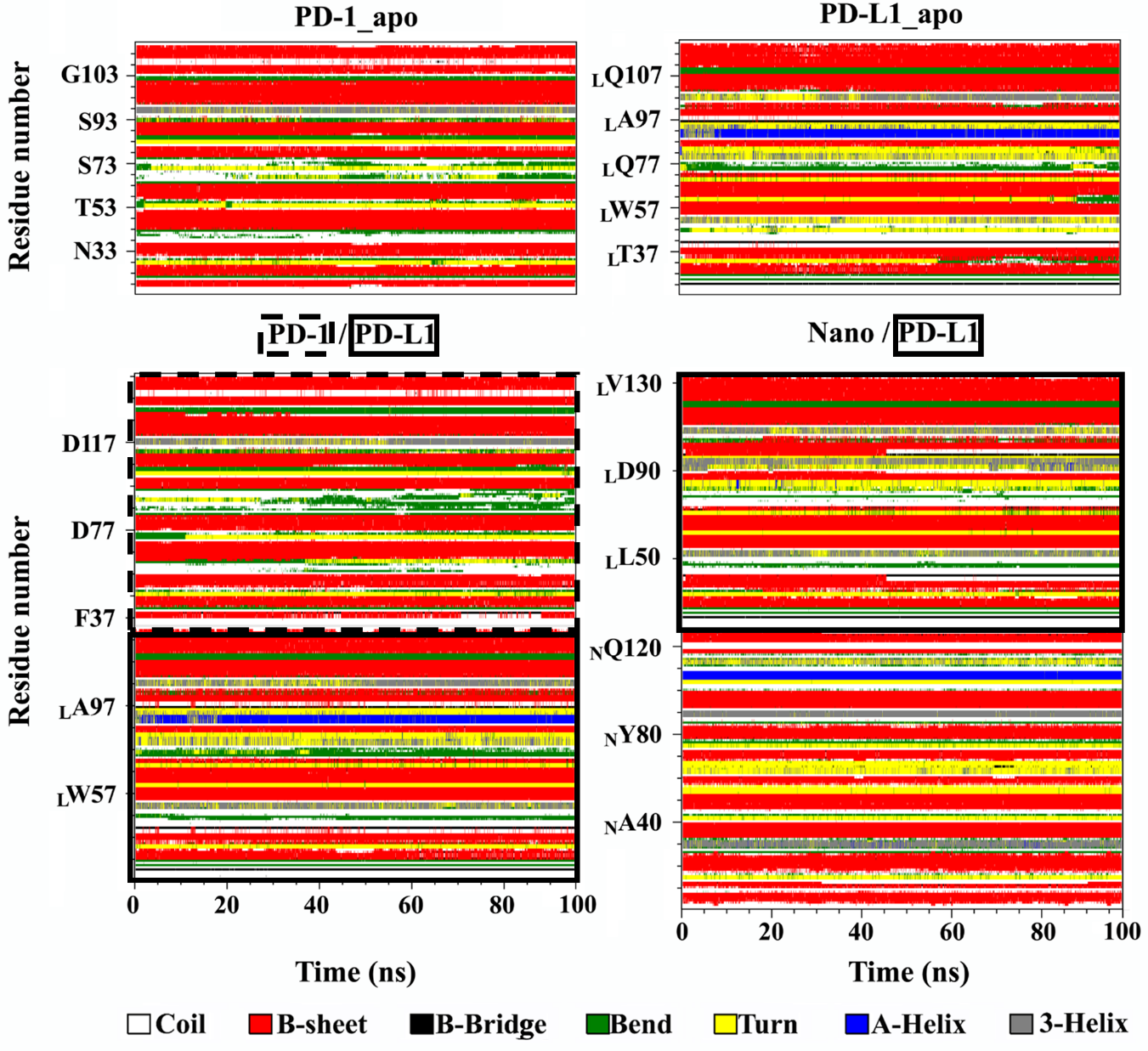


**Figure S5** The secondary structure of PD-1_apo, PD-L1_apo, PD-1/PD-L1 and Nano/PD-L1.


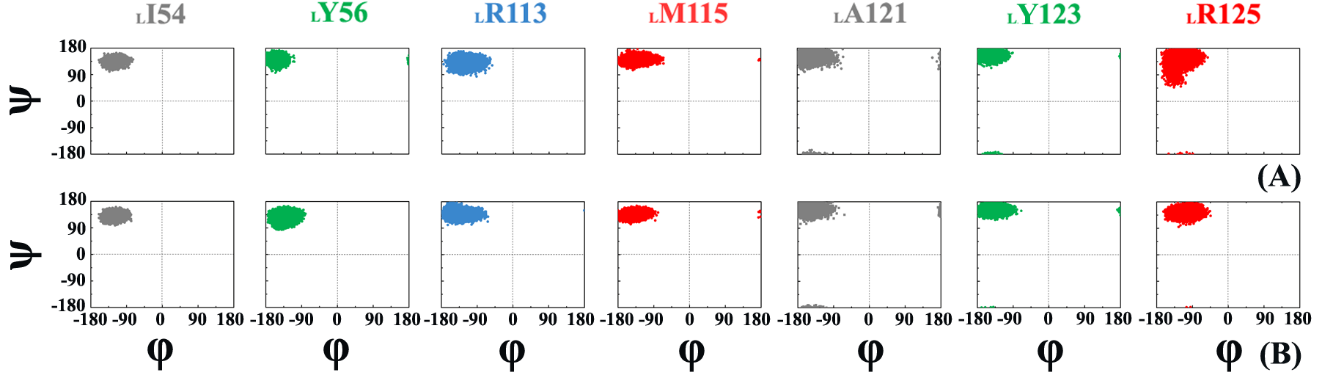


**Figure S6.** Two-dimensional distribution of dihedral angles in the β-sheet residues for the PD-1/PD-L1 (**A**) and Nano/PD-L1 (**B**) systems


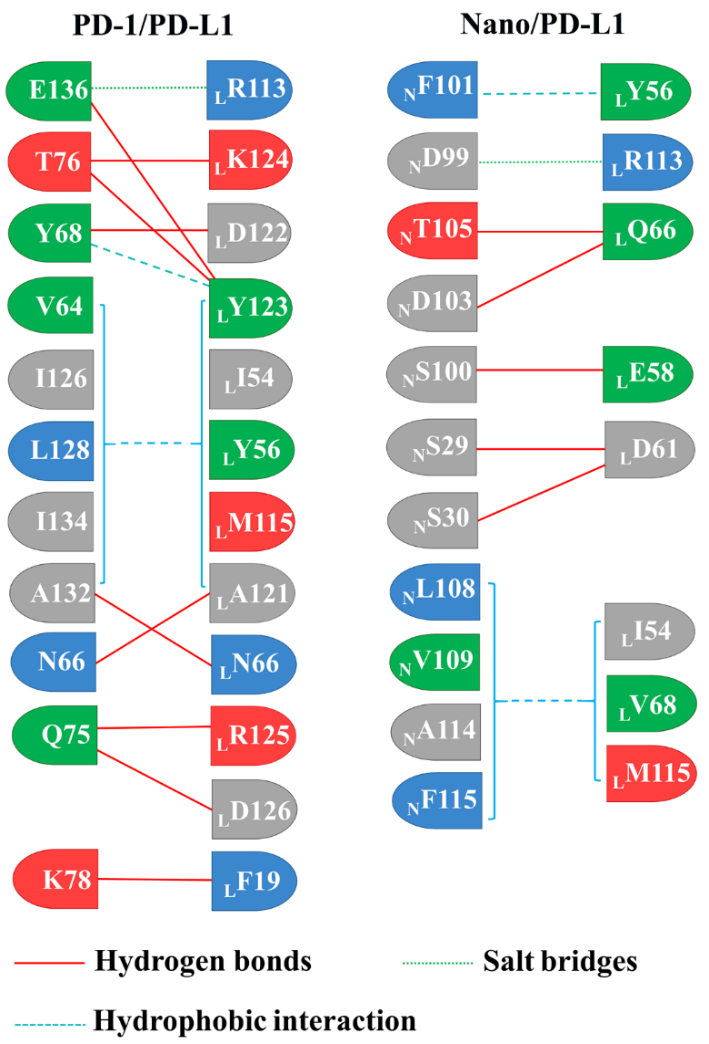


**Figure S7** The non-bonded interactions of PPI interface in PD-1/PD-L1 and Nano/PD-L1.


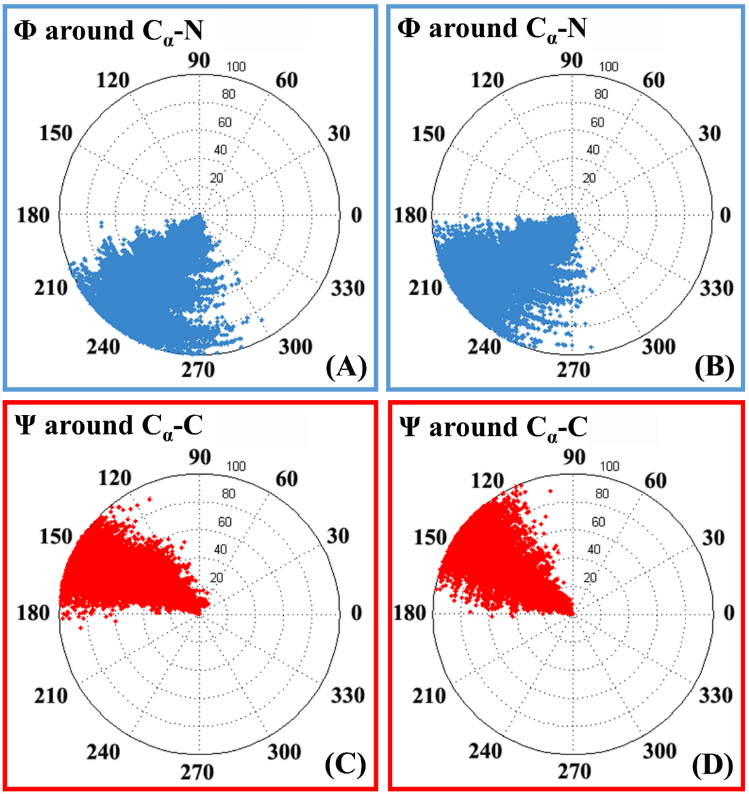


**Figure S8.** Dihedral angle change of _L_R113 (**A, B**) and _L_R125 (**C, D**)over time in PD-1/PD-L1 (**A, C**) and Nano/PD-L1 (**B, D**) systems.


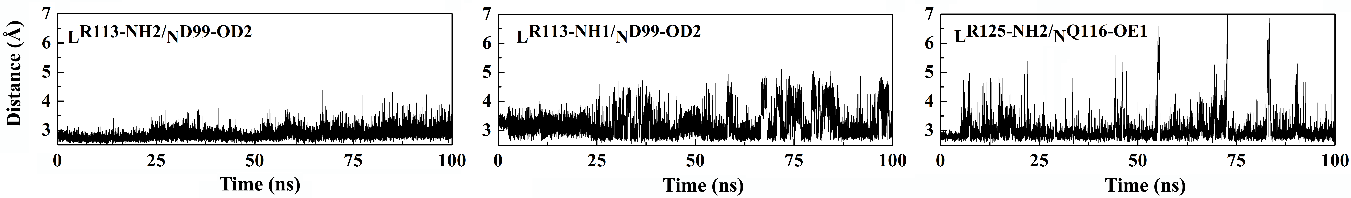


Figure S9 The distance’s variety of LR113-NH2/ ND99-OD2, LR113-NH1/ ND99-OD2 and LR125-NH2/ NQ116-OE1 over time.


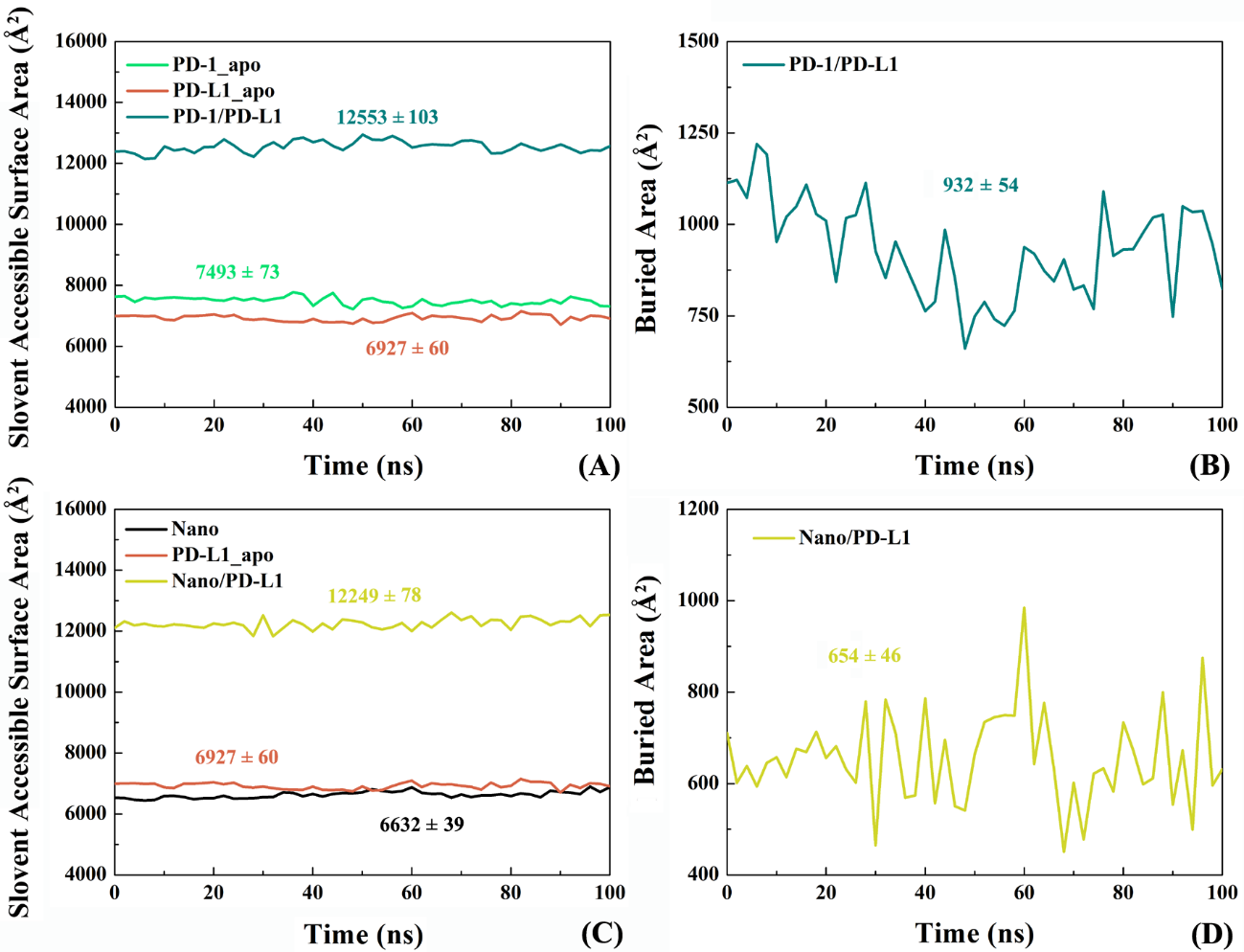


Figure S10 The variety of SASA in five simulation system (A/C), and the that of burried area in both complexes (B/D).
